# Compensation in Preclinical Huntington's Disease: Evidence From the Track-On HD Study

**DOI:** 10.1016/j.ebiom.2015.08.002

**Published:** 2015-08-04

**Authors:** Stefan Klöppel, Sarah Gregory, Elisa Scheller, Lora Minkova, Adeel Razi, Alexandra Durr, Raymund A.C. Roos, Blair R. Leavitt, Marina Papoutsi, G. Bernhard Landwehrmeyer, Ralf Reilmann, Beth Borowsky, Hans Johnson, James A. Mills, Gail Owen, Julie Stout, Rachael I. Scahill, Jeffrey D. Long, Geraint Rees, Sarah J. Tabrizi, the Track-On investigators

**Affiliations:** a Albert-Ludwigs-University Freiburg, University Medical Center, Division Freiburg Brain Imaging, Freiburg, Germany; b Albert-Ludwigs-University Freiburg, University Medical Center, Department of Psychiatry and Psychotherapy, Freiburg, Germany; c Albert-Ludwigs-University Freiburg, University Medical Center, Department of Neurology, Freiburg, Germany; d Wellcome Trust Centre for Neuroimaging, Institute of Neurology, University College London, London, UK; e Albert-Ludwigs-University Freiburg, Department of Psychology, Laboratory for Biological and Personality Psychology, Freiburg, Germany; f Department of Electronic Engineering, N.E.D University of Engineering & Technology, Karachi, Pakistan; g APHP Department of Genetics, Groupe Hospitalier Pitié-Salpêtrière, UPMC Université Paris VI UMR_S1127, Paris France; h Institut du Cerveau et de la Moelle, INSERM U1127, CNRS UMR7225, UPMC Université Paris VI UMR_S1127, Paris France; i Leiden University Medical Center, Department of Neurology, Leiden, The Netherlands; j Centre for Molecular Medicine and Therapeutics, Department of Medical Genetics, University of British Columbia, Canada; k Department of Neurodegenerative Disease, UCL Institute of Neurology, University College London, London, UK; l Department of Neurology, Ulm University, Ulm, Germany; m George-Huntington-Institute, Muenster, Germany; n University of Tuebingen, Department of Neurodegenerative Diseases and Hertie-Institute for Clinical Brain Research, Tuebingen, Germany; o CHDI Management/CHDI Foundation, Princeton, NJ, USA; p Department of Electrical and Computer Engineering, University of Iowa, Iowa City, IA, USA; q Department of Psychiatry, Carver College of Medicine, University of Iowa, Iowa City, IA, USA; r School of Psychological Sciences and Institute of Clinical and Cognitive Neuroscience, Monash University, Melbourne, Australia; s Department of Biostatistics, College of Public Health, University of Iowa, Iowa City, IA, USA; t Institute of Cognitive Neuroscience, University College London, London, UK

**Keywords:** Huntington's disease, Preclinical, Neural compensation, MRI, Cognitive, Motor

## Abstract

**Background:**

Cognitive and motor task performance in premanifest Huntington's disease (HD) gene-carriers is often within normal ranges prior to clinical diagnosis, despite loss of brain volume in regions involved in these tasks. This indicates ongoing compensation, with the brain maintaining function in the presence of neuronal loss. However, thus far, compensatory processes in HD have not been modeled explicitly. Using a new model, which incorporates individual variability related to structural change and behavior, we sought to identify functional correlates of compensation in premanifest-HD gene-carriers.

**Methods:**

We investigated the modulatory effects of regional brain atrophy, indexed by structural measures of disease load, on the relationship between performance and brain activity (or connectivity) using task-based and resting-state functional MRI.

**Findings:**

Consistent with compensation, as atrophy increased performance-related activity increased in the right parietal cortex during a working memory task. Similarly, increased functional coupling between the right dorsolateral prefrontal cortex and a left hemisphere network in the resting-state predicted better cognitive performance as atrophy increased. Such patterns were not detectable for the left hemisphere or for motor tasks.

**Interpretation:**

Our findings provide evidence for active compensatory processes in premanifest-HD for cognitive demands and suggest a higher vulnerability of the left hemisphere to the effects of regional atrophy.

## Highlights

• Huntington's disease (HD) is an ideal model for studying the earliest stages of neurodegeneration.
• Pre-clinical HD gene-carriers maintain normal behavior across motor and cognitive domains despite neuronal degeneration.
• We characterized neural compensation using functional MRI in combination with measures of disease load and task performance.
• Evidence of hemisphere-specific compensation provides potential targets for future therapeutic trials in neurodegeneration.

We have developed a model which characterizes compensation in preclinical HD using functional MRI, volumetric disease load and task performance. By explicitly testing the relationship between cognitive performance and brain activity or connectivity, we have demonstrated that those closest to clinical diagnosis experience functional deterioration within the left hemisphere of the brain and localized compensation in the right. This pattern provides potential targets for future testing of HD modifying agents. Furthermore, HD acts as a model for studying the earliest stages of neurodegeneration and this new model of compensation can potentially be extended to other neurodegenerative diseases.

## 1 Introduction

In neurodegenerative disease, progressive degenerative changes can be detected many years prior to the manifestation of clinical symptoms including cognitive and motor deficits. Such findings indicate that the brain has capacity to compensate for degenerative losses, maintaining normal levels of cognitive and motor function, until such time that neuropathology translates into clinical loss of function. For example, in Huntington's disease (HD), a fully penetrant monogenic disorder, individuals far from the onset of overt signs and symptoms demonstrate extensive neuroimaging evidence of subcortical and cortical atrophy. However, such HD expansion mutation carriers perform similarly to healthy controls on a wide variety of motor and cognitive tests and show minimal longitudinal change in performance (Tabrizi et al., 2011, Papoutsi et al., 2014).

We can postulate two different mechanisms that might be responsible for this dissociation between progressive structural pathology and minimal measurable phenotypical behavioral change. Inherent cognitive reserve may mitigate against the emergence of measurable phenotypical alterations before a threshold for functional degradation is exceeded. Alternatively, secondary compensatory mechanisms, in which brain pathology leads to adaptation of neural activity patterns, may emerge in the course of the disease process and could create alternative or modified neural processes to support maintenance of cognitive and motor function at normal levels. However, no universally accepted definition of compensation exists (Barulli and Stern, 2013), and the underlying mechanisms are unknown.

Our previous longitudinal multi-site study (Track-HD) showed disease-related reductions in striatal and white matter volume (Tabrizi et al., 2009), and elevated rates of atrophy from the very earliest premanifest stages (Tabrizi et al., 2011, Tabrizi et al., 2012, Tabrizi et al., 2013). Despite this consistent progressive structural loss, high levels of functional performance are maintained in this cohort and there is little evidence of cognitive or motor decline over time (Tabrizi et al., 2009, Tabrizi et al., 2011, Tabrizi et al., 2012, Tabrizi et al., 2013). The current study (Track-On HD) was designed to explore the hypothesis that compensatory brain networks exist to maintain function in the presence of widespread structural damage during the premanifest phase of HD.

**Fig. 1:**
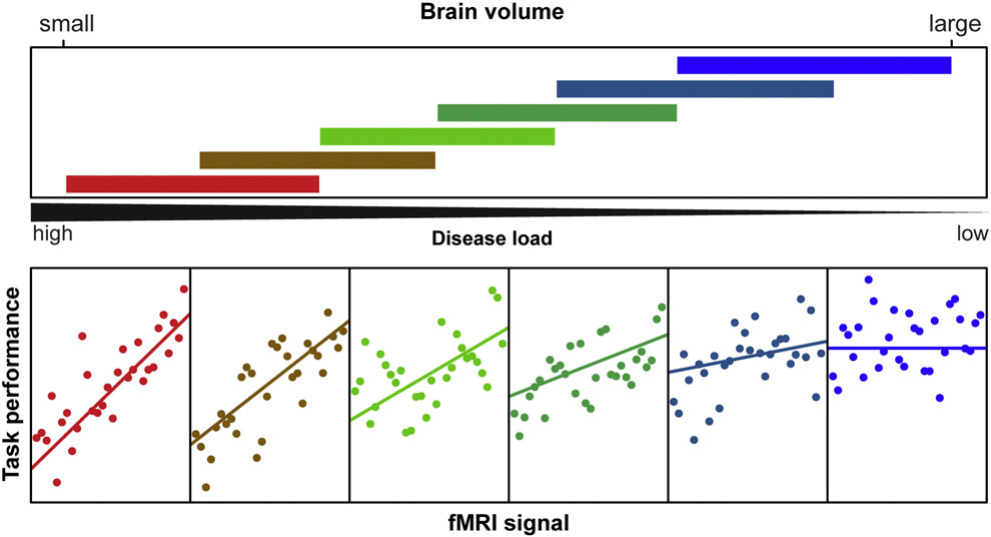
Example conditioning plot with simulated data. The upper panel depicts the overlapping ranges of structural disease load as measured by brain volume (the slabs) that determine the subsample for each scatterplot panel below. Observed points are plotted in the lower panel scatterplots for each range of brain volume, and a linear regression line is fit separately in each panel to aid interpretation. The structural disease load (brain volume) range determines what subsample is selected from the data set for the scatterplot of task performance as a function of fMRI signal, with the color coding showing the correspondence. For example, the extreme left scatterplot (red) includes the smallest brain volume (highest disease load) range from the data set (lower left red slab). The extreme right scatterplot (blue) includes the largest volume (lowest disease load) range from the data set (upper right blue slab). Of note, regression lines and the separation in different slabs have illustrative purposes only and are not the basis of the underlying statistic.

Recent functional neuroimaging studies indicate augmented task-related brain activity in premanifest HD expansion mutation-carriers compared with healthy control or manifest HD groups, (Georgiou-Karistianis et al., 2013, Gray et al., 2013, Klöppel et al., 2009, Novak et al., 2012, Poudel et al., 2013, Scheller et al., 2013, Wolf et al., 2007, Malejko et al., 2014) providing evidence of increased subcortical (Georgiou-Karistianis et al., 2013, Malejko et al., 2014), and cortical activation in both prefrontal and parietal cortex as well as supplementary motor areas (Klöppel et al., 2009, Scheller et al., 2013). Typically, published studies using such comparisons neither take into account disease-related structural alterations within the groups, nor the variability in performance within each group. Moreover, using group comparison leaves open the possibility that the observed differences in brain activity are not task-related but instead reflect a superimposed effect of neurodegenerative pathology. To overcome these challenges, we developed a measure of neural compensation that takes into account the relationships between behavioral performance and brain activity (or connectivity) seen in healthy individuals, and applied it to functional MRI (fMRI) measures of brain activity in a large cohort of over 100 individuals with premanifest-HD (preHD). We hypothesized that neural compensation could be identified as a positive change in the relationship between performance and brain activation in association with relatively high levels of structural alterations reflecting the impact of the disease processes (‘structural disease load’). Specifically, we hypothesized that for those brain regions showing compensation, higher structural disease load would be associated with tighter relationships between performance and brain activity (Fig. 1 and Eq. (1), Materials and Methods) indicating a need for greater task-associated neural activity to maintain similar levels of performance in individuals with higher structural disease load.

We characterized structural disease load using volumetric measures of the caudate, putamen, global gray and global white matter that show sensitivity towards HD-related changes (Tabrizi et al., 2012). These structural markers of disease load were included in a systematic examination of both brain activity and connectivity. Brain activity was measured during performance of a motor or cognitive task using task-fMRI and brain connectivity within cognitive and motor networks using resting-state fMRI (rsfMRI). This allowed us to perform a comprehensive, unbiased whole-brain assessment of potential markers of compensation for neurodegeneration in preHD.

## 2 Materials and Methods

### 2.1 Participants

239 participants were recruited from the four Track-On HD study sites and comprised the following three groups: (1) 106 individuals without HD but carrying the mutant huntingtin gene (F 54; mean age ± SD: 42 · 80 ± 9 · 10), (2) 22 early HD patients (F 15, mean age ± SD: 45 · 22 ± 7 · 89), and (3) 111 age- and sex-matched controls (F 67, mean age ± SD: 48 · 14 ± 10 · 70). The 22 early HD patients were removed from all analyses as very few participated in all assessments. 14 participants were left-handed and were excluded from the motor fMRI task and resting state analyses (Oldfield, 1971). Most preHD and control participants were recruited from the Track-HD study (Tabrizi et al., 2009). Newly-recruited preHD participants were required to have a CAG repeat length ≥ 40 and a disease burden score greater than 250 at recruitment (Penney et al., 1997). Newly recruited control participants were either the partner or spouse of a participant, not at risk of HD, or HD normal-repeat-length sibling or control volunteers. For both groups, exclusion criteria included manifest disease, age below 18 or above 65 (unless previously in Track-HD study), major psychiatric, neurological or medical disorder or a history of severe head injury (see Supplementary Data). The study was approved by the local ethics committees and all participants gave written informed consent according to the Declaration of Helsinki.

### 2.2 Power Calculation

The current Track-On study was meant as an exploratory extension of the highly successful Track-HD study. The main purpose was to generate high resolution structural and fMRI data to explore issues such as compensation. Because such data were not previously generated from Track-HD, and compensation was not examined, there was no principled means of estimating required sample size. Now that these data have been collected and analyzed, sample size calculation is possible for future studies.

### 2.3 Behavioral Measures

A principal component analysis was performed on nine cognitive assessments to derive a global cognitive composite score (see Supplementary Data). Based on the Track-HD study (Tabrizi et al., 2013), the Unified Huntington's Disease Rating Scale (UHDRS)–Total Motor Score (TMS), and Grip Force Variability (GFV) were selected as markers of motor performance in both the task and rsfMRI analyses as both are sensitive to HD-related change (see Supplementary Data) Reilmann et al., 2010, Group, 1996.

### 2.4 fMRI Tasks

#### 2.4.1 Verbal Working Memory (VWM) Task

**Fig. 2:**
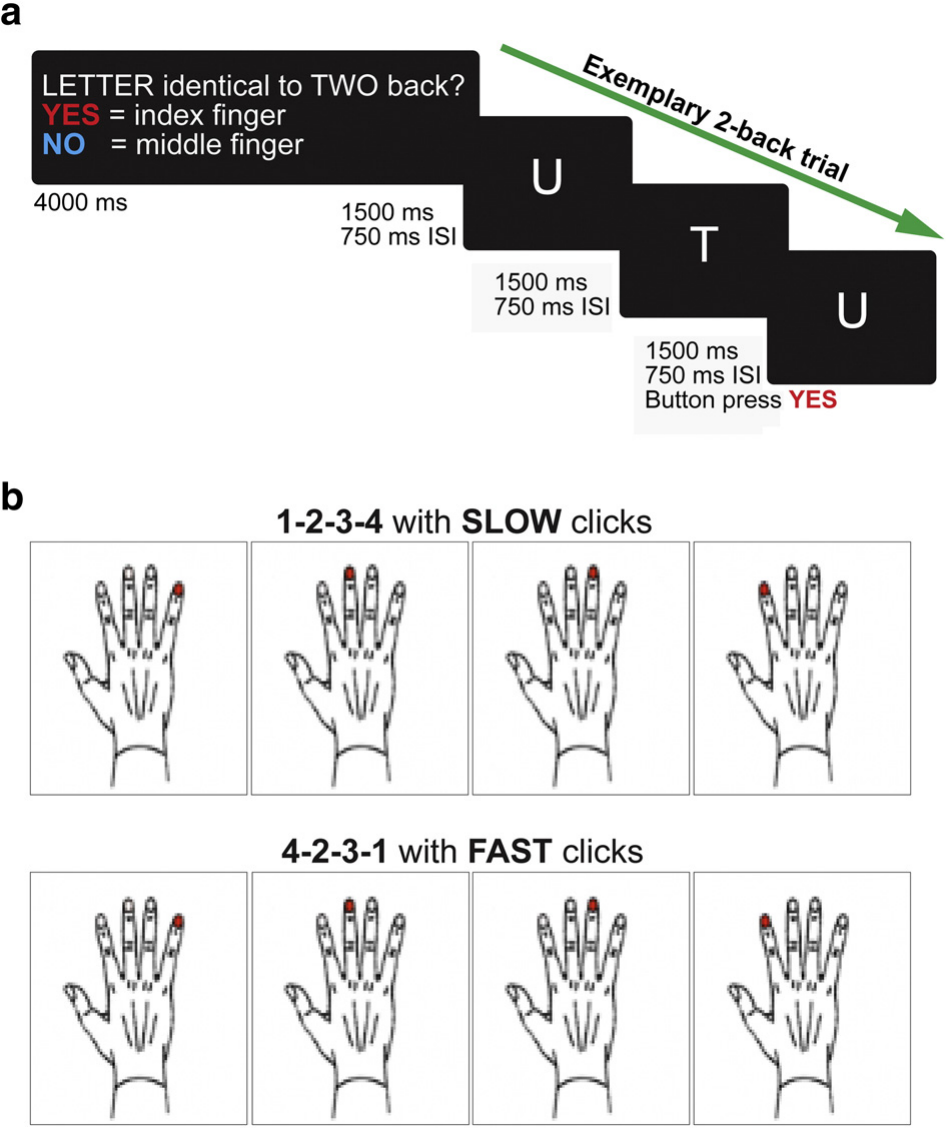
Task fMRI paradigms. Example trials for a) the verbal working memory n-back task and b) sequential finger tapping. Please see Materials and Methods for further details.

Participants performed a VWM n-back task with two levels of working memory load (1-back and 2-back) (Fig. 2). During the task, letters were presented one-by-one and participants were required to respond according to whether the letter on screen was the same as the letter presented one letter previously (1-back) or presented two letters previously (2-back) using a button box. A third condition (0-back) whereby participants indicated whether the letter A or B was presented on screen served as a baseline measure (see Supplementary Data). Performance in the 1-back and 2-back conditions was analyzed using both the *d*-prime coefficient (probability of correct response minus probability of false positive responses) and reaction times and assessed across groups and conditions using a repeated measures ANOVA adjusting for age, gender, site and education.

#### 2.4.2 Sequential Finger Movement (SFM) Task

Participants performed a motor task that involved metronome-paced finger tapping with their right (dominant) hand (see Klöppel et al., 2009 for a detailed description; Fig. 2). Speed and complexity of the tapping sequence were varied to test executive (speed) and cognitive (complexity) demands of the task (see Supplementary Data). Mean timing inaccuracies (cue-response intervals) and standard deviations were analyzed using a 2 × 2 × 2 repeated measures ANCOVA, with complexity (simple or complex) and speed (slow or fast) as within-subject factors and group as a between-subject factor, adjusting for age, gender, site and education.

### 2.5 MRI Data Acquisition

3T MRI data was acquired on two different scanner systems (Philips Achieva at Leiden and Vancouver and Siemens TIM Trio at London and Paris), as described (Tabrizi et al., 2009). For task and rsfMRI, whole-brain volumes were acquired at a repetition time of 3 s using a T2*-weighted echo planar imaging (EPI) sequence with the following parameters: TE 30 ms, FOV 212 mm, flip angle 80°, 48 slices in ascending order (slice thickness: 2 · 8 mm, gap: 1 · 5 mm, in plane resolution 3 · 3 × 3 mm) and bandwidth of 1906 Hz per Px. Rs-fMRI data were collected first, then both sets of task fMRI data. For rs-fMRI, 165 volumes were acquired over 8:20 min followed by field map acquisition. 225 volumes over 11:15 min for the SFM task and 190 volumes over 9:30 min of VWM task fMRI data. Field maps were acquired with TR 1020 ms, TE1 10 · 0 ms, TE2 12 · 46 ms, FOV 212 mm and 2 mm slice thickness. All data were visually inspected by IXICO. Standardisation of data acquisition across sites was performed based on previous suggestions (see Supplementary Data) (Glover et al., 2012).

### 2.6 MRI Data Processing

T1-weighted images were processed as described (Tabrizi et al., 2009). Regional volumes were adjusted for intracranial volume (ICV) and used as structural disease load measures for compensation analyses. fMRI data preprocessing and subsequent statistical analyses were performed using SPM8 running under MATLAB. The T1 scan was segmented into gray and white matter using the VBM8 toolbox (http://dbm.neuro.uni-jena.de/vbm/) and used to create an improved anatomical scan for coregistration. Using the DARTEL extension, deformation parameters were extracted for normalization of functional images (Ashburner, 2007). The first four EPI images were discarded to allow for steady state equilibrium. Functional images were first realigned and field maps used for inhomogeneity correction whenever available. For rsfMRI, all EPI images were then coregistered to the new anatomical image and normalized using DARTEL deformation parameters. For task fMRI, only contrast images were normalized. Finally, data were smoothed using a 6 mm full width at half maximum Gaussian kernel. Although for task fMRI a smoothing kernel of 8 mm is more conventional, in the current study we used a kernel of 6 mm for consistency between rsfMRI and task fMRI data (for Quality Control see Supplementary Data).

### 2.7 MRI Data Analysis

#### 2.7.1 Task fMRI Data Specific Analyses

A first-level analysis based on the general linear model (GLM) was performed for each participant on the smoothed images. Task-related BOLD signal changes were estimated for each task condition. Six movement regressors were also modeled, in addition to the instruction screen, single button presses during rest and blocks during which participants performed a wrong condition for the motor task (for Group Analysis see Supplementary Data). The 2-back vs 1-back contrast (VWM) and complexity (complex > simple) and speed (fast > slow) contrasts (SFM) were used to identify the significant voxels (p < 0.05 FWE-corrected) which entered the whole brain compensation analyses described below. In the compensation model, findings are reported at p < 0 · 001 without correction for multiple comparisons.

#### 2.7.2 Resting State fMRI Analysis

Resting state fMRI data is used to interrogate task-positive networks including, among others, motor, attention and executive function networks, in the brain at rest Beckmann et al., 2005, Smith et al., 2012. Resting state fMRI data were analyzed using two complementary connectivity analysis techniques. Seed-region based correlation (functional connectivity) was used to investigate temporal correlations between activity within a region specific to a network of interest and the whole brain. Dynamic Causal Modeling (DCM; effective connectivity) probed causal influence between a number of network-relevant regions within a pre-defined model (Li et al., 2011).

##### 2.7.2.1 Functional Connectivity Analyses

Seed regions of interest for the cognitive network were located in the left and right Dorsolateral Prefrontal Cortex (DLPFC) (Owen et al., 2005), and for the motor network within the left primary motor cortex (M1). The time series for each seed region was extracted from the smoothed scans using a 4 mm radius sphere, centered on the seed region co-ordinates (see Supplementary Data). The extracted time-series was then entered into a GLM which also included the representative non-neuronal time-series for both white matter and CSF signal (extracted as above) and six movement regressors. The individual correlation maps for each seed region analysis were included in a one-tailed, one sample *t*-test of all participants. Parameter estimates were extracted from voxel clusters with significant positive correlations (p < 0 · 05 FWE-corrected) using the SPM toolbox MARSbar v0 · 43 (http://marsbar.sourceforge.net/) and entered our compensation model.

##### 2.7.2.2 Effective Connectivity Analyses

**Fig. 3:**
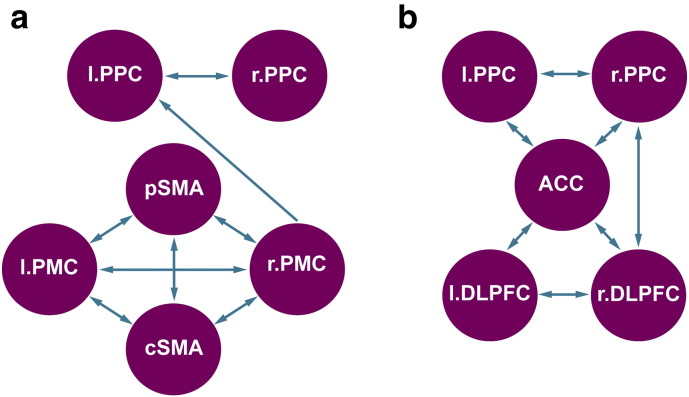
Dynamic causal models employed for resting state fMRI. a) Motor network and b) cognitive network. Abbreviations: ACC: anterior cingulate cortex; DLPFC: dorsolateral prefrontal cortex; PMC: premotor cortex; PPC: posterior parietal cortex; and SMA: supplementary motor area.

Regions for the network models were derived from the task-fMRI analyses (Fig. 3) and all biologically plausible connections modeled (see Supplementary Data). The time-series for each region was extracted from all voxels within an 8 mm radius sphere centered on the co-ordinates using a GLM that included white matter and CSF signal in addition to movement regressors. Cognitive and motor networks were then modeled using DCM specification and estimation carried out with DCM10 in Statistical Parametric Mapping software (SPM12b; Wellcome Trust Centre for Neuroimaging, http://www.fil.ion.ucl.ac.uk/spm). Resultant effective connectivity parameters were entered into a one-sample *t*-test of all participants and significant connectivity values (FDR-corrected) extracted and examined within our compensation model.

### 2.8 Models of Compensation

Fig. 1 is a conditioning plot that illustrates the basic concept of compensation. The crux of compensation is that the relationship between a response variable (cognitive or motor) and an fMRI variable is conditional on (or varies by) structural disease load (brain volume). Underlying this concept is the statistical interaction among structural disease load and the fMRI variable in question. The compensation model was defined as the following. Suppose a performance measure is denoted as *y*, structural disease load is denoted as *d*, fMRI-signal is *f*, and a vector of covariates is ***c***. Then the statistical model can be written as

(1) $$y=\alpha +\beta_{1}d+\beta_{2}f+\beta_{3}\left(d\right)\left(f\right)+\gamma c+e$$

where *α* is the intercept or offset, the *β*_1_ and *β*_2_ are main effects, *β*_3_ is the interaction term between structural disease load and fMRI activity, ***γ*** is a vector of regression coefficients for the covariates, and *e* is random error (assumed to be normally distributed with zero mean and non-zero variance). Evidence for compensation was provided by the rejection of the null hypothesis, *H*_0_ : *β*_3_ = 0, which meant that the relationship between *f* and *y* varied significantly by *d* (cf. Fig. 1). Structural disease load was represented by caudate, putamen, white matter or gray matter volume as a fraction of total intracranial volume. These volumes were analyzed one at a time. To control for potential confounding effects, ***γc*** adjusted for age, gender, study site, education level, and cumulative probability of onset (CPO), the latter being included to account for current disease status. fMRI-signal either represented task-specific activations or functional or effective connectivity for the rsfMRI analyses.

Visualization of interactions was accomplished using conditioning plots (Fig. 1). The coplot consists of two types of panels. The upper panel consists of slabs that show the overlapping ranges of the structural disease load conditioning variable that dictates which observations from the sample are selected for illustration. The intervals of the conditioning structural disease load variable have the properties that approximately the same number of observations lies in each interval and approximately the same number of observations lies in two successive intervals (Chambers, 1992). The lower panels show the scatterplot of the selected observations for the performance variable (vertical axis) and the fMRI variable (horizontal axis). For illustrative (and not inferential) purposes, a linear regression line is fit to each scatterplot separately. The interior scatterplot panels illustrate the changing relationship between performance and fMRI activation over part of the range of the structural disease load variable.

For task fMRI data, the compensation model was applied to all voxels individually within the respective task-specific main effect. For the rsfMRI-based connectivity analyses, the compensation model was applied to the average signal value extracted from each region significantly correlated with seed regions and each significant connection from the respective cognitive and motor network DCM. As predicted markers of performance, for task fMRI, we used behavioral data obtained while performing the VWM and SFM tasks in the scanner. For rsfMRI data the global cognitive composite score, GFV and UHDRS-TMS were used.

Alpha-adjustment was not used in the multiple tests of *H*_0_ : *β*_3_ = 0 for the following reasons. Neuroscience meta-analysis indicates that effects in the field tend to be small and the statistical power tends to be low in general (Button et al., 2013, Uttal, 2013). Compensation effects are expected to be especially small because they are expressed in an interaction term among correlated predictors, as shown in Eq. (1). The compensation interaction must show an effect over and above the main effects with which it is correlated, perhaps highly so. Given the compensation definition of Eq. (1) and the fact that the study was not powered to detect compensation, the decision was made to use the nominal p ≤ 0 · 05 criterion for statistical significance for all compensation tests. The justification was the ability to detect relatively fragile compensation effects that would probably never endure alpha-adjustment with such a small sample size. The lack of adjustment does provoke strong caution regarding the interpretation of the results. p-Values close to 0 · 05 might require very large sample sizes for replication and can have spuriously inflated effect sizes (Colquhoun, 2014).

## 3 Results

### 3.1 Cognitive Network

#### 3.1.1 VWM Task fMRI

**Fig. 4:**
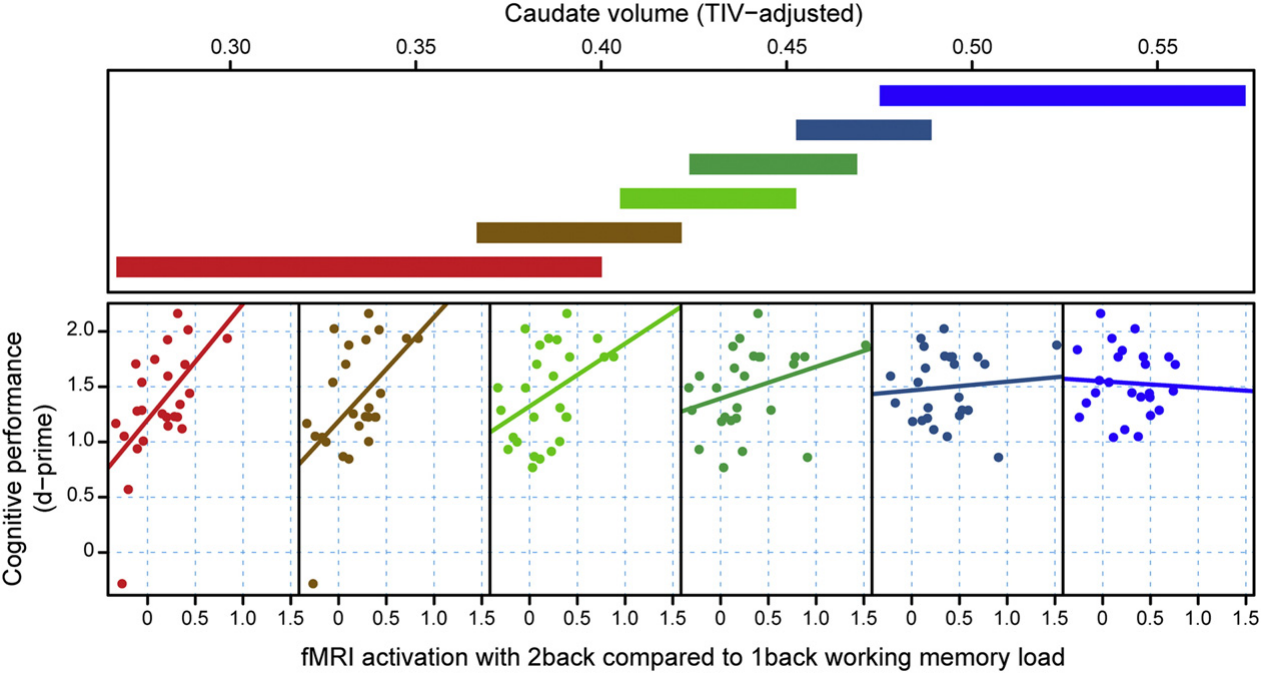
D-Prime performance as a function of fMRI task activation within the parietal cortex, conditional on caudate volume as a measure of structural disease load. For each plot, the upper panel depicts the overlapping ranges of caudate volume that determine which subsample is selected from the data set that is used to construct each scatterplot. A linear regression line was fit for each scatterplot to aid interpretation.

For behavioral and main effect fMRI analyses see Supplementary Data. We performed whole brain compensation analyses separately for each voxel significant for the main effect of VWM (p < 0 · 05, corrected). The compensation interaction was statistically significant for the right superior parietal cortex (x = 39, y = − 60, z = 45; T = 3 · 47, p < 0 · 002) and the inferior parietal cortex (x = 38, y = − 54, z = 29; T = 4 · 18, p < 0 · 001). This relationship between VWM-task performance and the parietal cortex activity conditional on structural disease load (caudate volume) is visualized in Fig. 4. There was a relatively strong and positive relationship between VWM-task performance and cortex activity for high structural disease load, but the relationship diminished as structural disease load lightened with the lowest structural disease load showing no relationship. Similar results were also evident when using putaminal volume, but not white or gray matter as markers of structural disease load.

#### 3.1.2 Resting State fMRI — Functional Connectivity and Compensation

**Fig. 5:**
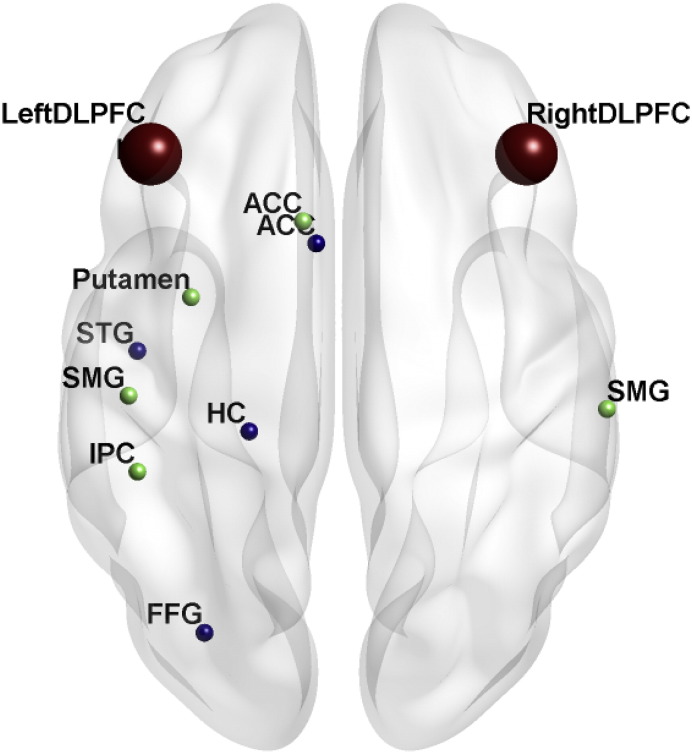
Overview of functional connectivity analyses for the cognitive network. Regions that significantly correlated (p < 0.05 FWE-corrected) with seed regions in the right (blue) or left DLPFC (green) and which also, as part of the compensation model, significantly predicted global cognitive performance as structural disease load increased. Abbreviations: ACC: anterior cingulate cortex; DLPFC: dorsolateral prefrontal cortex; FFG: fusiform gyrus; HC: hippocampus; IPC: inferior parietal cortex; and SMG: supramarginal gyrus. STG: superior temporal gyrus.

**Fig. 6:**
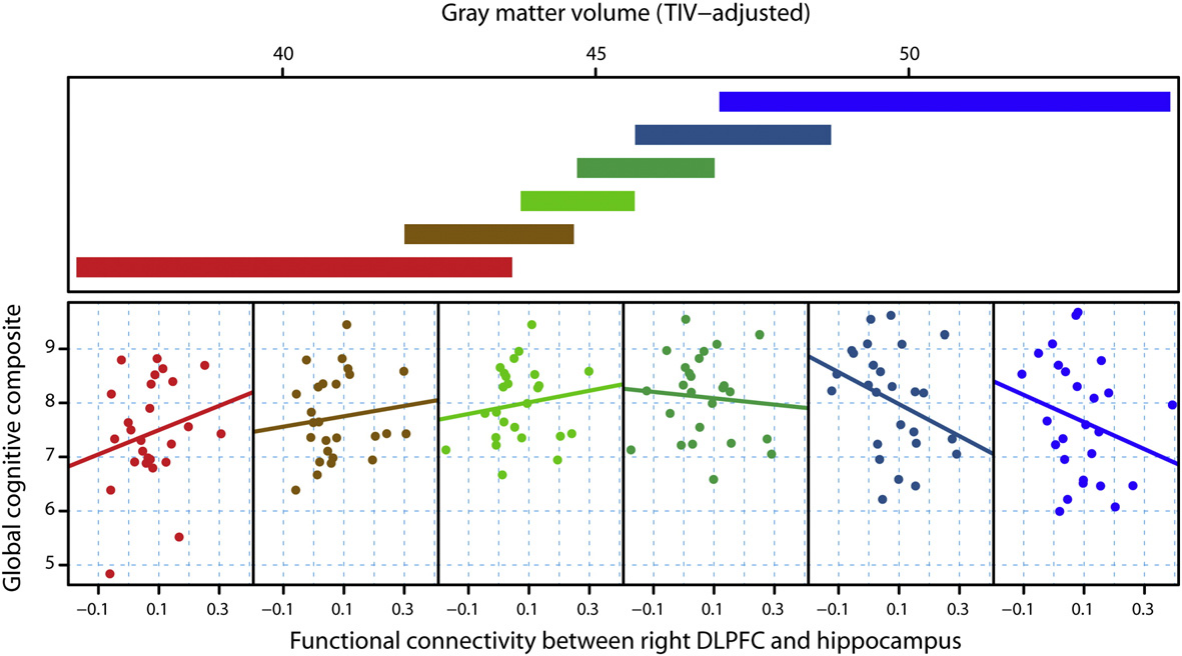
Global cognitive performance as a function of rsfMRI functional connectivity between right DLPFC and left hippocampus, conditional on gray matter volume as a measure of structural disease load. For each plot, the upper panel depicts the overlapping ranges of gray matter volume that determine which subsample is selected from the data set that is used to construct each scatterplot. A linear regression line was fit for each scatterplot to aid interpretation.

For the right DLPFC seed, this analysis revealed that functional connectivity between the right DLPFC and a distributed set of regions in the left hemisphere (Fig. 5, Fig. 6; Fig. S3; Table S3) exhibited a statistically significant interaction between coupling and disease load in the prediction of global cognitive performance. As seen in Fig. 6, the relationship between coupling and global cognitive performance increased as structural disease load increased, consistent with our operational definition of compensation, but decreased in those furthest from onset (with the lowest structural disease load). The regions whose coupling with right DLPFC showed such a relationship included the fusiform gyrus (FFG) (p = 0 · 010), the inferior frontal gyrus (p = 0 · 019), the hippocampus (p = 0 · 034), the superior temporal gyrus (p = 0 · 049), and the anterior cingulate cortex (ACC) (p = 0 · 051); however, only the FFG survived the pre-planned Bonferroni-correction for structural disease load. The first three findings were observed using gray matter volume as the measure of structural disease load, and the latter two using putamen volume. In all these regions, the correlations between global cognitive performance and functional connectivity that demonstrated increased slope with structural disease load suggest a compensatory effect in the right hemisphere in preHD.

**Fig. 7:**
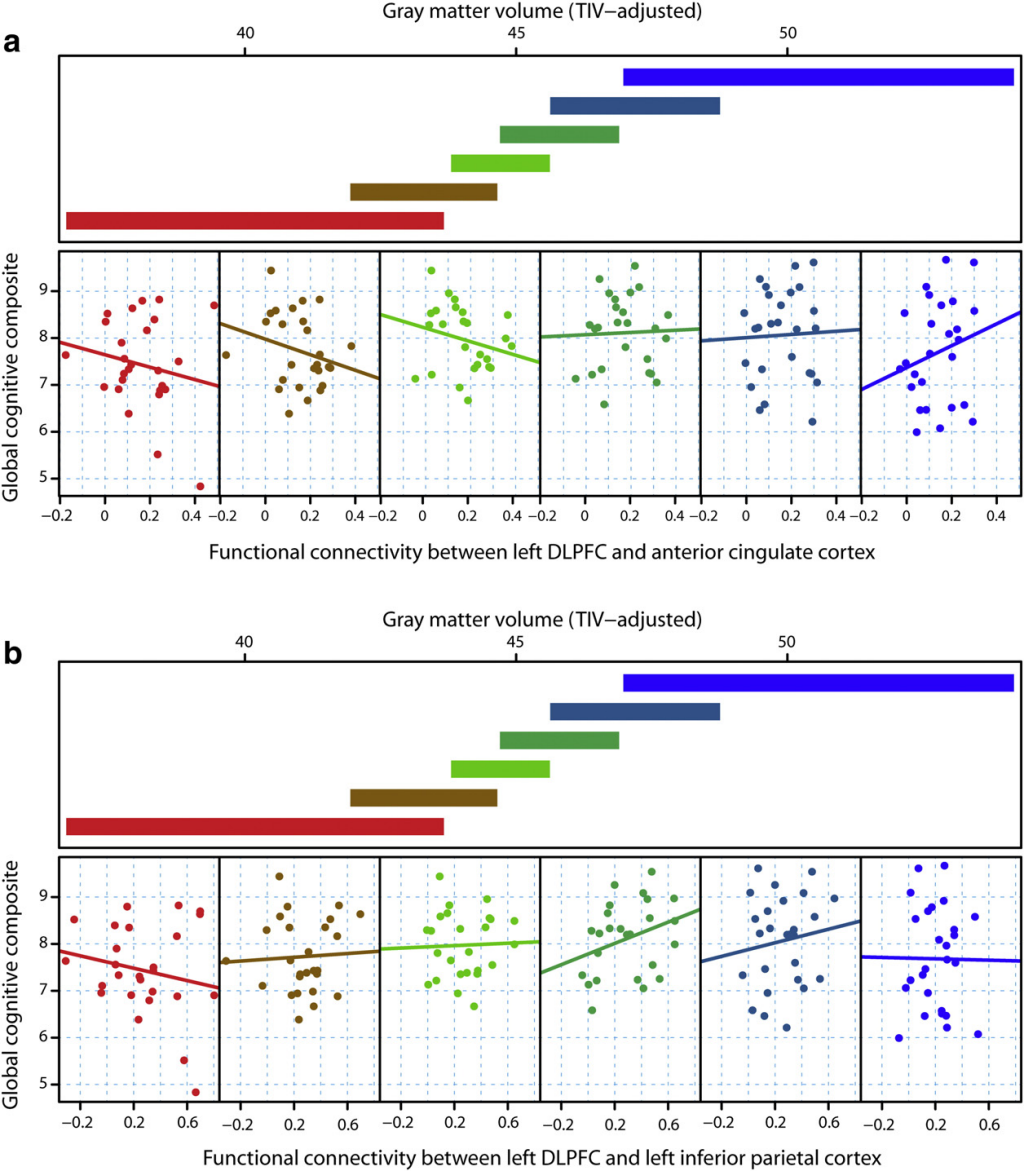
Global cognitive performance as a function of rsfMRI functional connectivity between left DLPFC and a) anterior cingulate cortex, or b) left inferior parietal cortex, conditional on gray matter volume as a measure of structural disease load. For each plot, the upper panel depicts the overlapping ranges of gray matter volume that determine which subsample is selected from the data set that is used to construct each scatterplot. A linear regression line was fit for each scatterplot to aid interpretation.

In contrast to the right DLPFC, we found that functional connectivity between the left DLPFC and a number of regions that was associated with global cognitive performance had a negative relationship that varied by structural disease load (Fig. 5, Fig. 7; Table S4). These regions included the left inferior parietal cortex (p = 0 · 007), ACC (p = 0 · 004) and the left supramarginal gyrus (SMG) (p = 0 · 003), all of which survived Bonferroni-correction for structural disease load, in addition to correlations with the left putamen (p = 0 · 01) and right SMG (p = 0 · 042), which did not survive Bonferroni-correction. All correlations were observed using gray matter volume as a measure of structural disease load; with the inferior parietal cortex also significant when using white matter as a marker of structural disease load. In all these regions, we therefore found relationships between resting-state brain activity, structural disease load and global cognitive performance that, according to our definition of compensation, argue against compensatory effects being evident in the left hemisphere for preHD.

#### 3.1.3 Resting State fMRI — Effective Connectivity and Compensation

We did not find any DCM connectivity parameters that were correlated with global cognitive performance and changed with disease load in a fashion predicted by our compensation hypothesis (see Supplementary Data).

### 3.2 Motor Network

#### 3.2.1 SFM Task fMRI

For behavioral and main effect analyses see Supplementary Data. The compensation model did not reveal any significant relationship between speed and complexity (as performance markers), brain activity and structural disease load. This remained the case even when using an exploratory threshold of p < 0 · 01 uncorrected.

#### 3.2.2 Resting State fMRI — Functional Connectivity and Compensation

In the first step of this analysis, we did not find any regions in which activity significantly (p < 0 · 05, corrected) correlated with that of the left M1. Therefore, no further compensatory analyses of functional connectivity for the motor network were conducted.

#### 3.2.3 Resting State fMRI — Effective Connectivity and Compensation

Findings are indicative of a non-compensatory effect in both hemispheres in those preHD close to onset (see Supplementary Data).

## 4 Discussion

We used a new approach to investigate and characterize at a systems level neural compensation in preHD, by characterizing the relationship between brain activity and task performance, conditional on structural disease load. We combined task and rsfMRI with structural MRI-derived volumetric measures of disease load, and detailed clinical, cognitive, and motor assessment, and identified a potential compensatory network in which increases in right-hemisphere activation and connectivity predicted better preserved cognitive performance in HD expansion mutation-carriers with higher structural disease load.

Investigation of specific regions associated with performing a VWM task and modulated by task difficulty revealed progressively more positive correlations between activity in a region of the right parietal cortex and better detection accuracy during the VWM task in those approaching disease onset (compared to those further from disease onset). Complementing these findings, a simple seed-based functional connectivity approach using resting state data identified a network of compensatory connections focused in the right hemisphere. These patterns of connectivity in the resting state demonstrate a relationship between task performance and activity that changed with disease load in a manner consistent with our operational definition of compensation.

In contrast, brain activity and connectivity centered in the left hemisphere showed no evidence of compensatory changes in activity. A functionally more resilient right hemisphere is consistent with findings from previous studies (Lambrecq et al., 2013, Muhlau et al., 2007), which have already indicated that HD-pathology is in a subtle, but robust fashion, leftward biased. Use and stress-related neuronal demands, potentially including excitotoxic mechanisms, may be more pronounced in the dominant left hemisphere and may underlie asymmetry (Jenkins et al., 1998). The notion of a subtle but reproducible larger left-hemispheric deterioration in HD requires further investigation in terms of the pathophysiological underpinnings of this asymmetry. For example, these changes could be related to a more metabolically active left hemisphere with higher energy demands (Mochel et al., 2012), thus making it more susceptible in HD, in which bioenergetic defects are well documented (Ross and Tabrizi, 2011). Alternatively, it may reflect a use-dependent possible prion-like spread of mutant huntingtin resulting in more subtle, but extensive damage in the left hemisphere in a functional connectivity-dependent fashion (Ross et al., 2014).

It is surprising that despite evidence of compensation in cognitive networks associated with working memory performance, neither the task-based nor the rsfMRI-based effective connectivity analyses provided evidence of a compensatory mechanism in the motor system. For task-based fMRI, this could potentially be due to the use of a motor task that was insufficiently challenging to participants to engage compensatory processes. Of note, a SFM task previously studied in preHD with more demanding difficulty levels than those used in the current study revealed some evidence of compensation Klöppel et al. (2009). In this study, participants were required to memorize an irregular 10-item sequence of finger movements. Here, we aimed to remove the working memory component from the SFM task and replace it with an independent working memory task. Importantly the compensation model used in the previous study did not consider differing levels of performance within preHD and healthy participants, as did the current compensation model. Differences between the studies could therefore relate to the precise compensation model used, emphasizing the importance of operationally and explicitly defining neural compensation. Our rsfMRI-based analysis of functional coupling between key regions of the motor system did not provide any additional insights, as no regions were sufficiently correlated with the seed region to enter compensation analyses. Based on previous evidence, we did expect to see correlations between activity within the M1 and that of other regions of the brain, particularly regions within the motor network. We did identify significant correlations, but these were present at lower thresholds. Given that our thresholds were defined *a priori* and that the connectivity parameters were simply extracted for inclusion in our compensation model, we were unable to report lower threshold findings. For future longitudinal analyses, we will modify our approach to include regions of the motor network that are temporally correlated.

DCM-based markers of effective connectivity between pre-specified regions of the motor network also did not indicate any compensation. However, it should be noted that DCM is limited by the necessity to specify regions *a priori*, and untested compensatory mechanisms involving brain areas not incorporated in the pre-defined list of regions of interest would remain undetected. In addition, a compensatory mechanism may become apparent only at a time window very close to the emergence of phenotypical motor abnormalities or may not be operative at all for the motor system. This is consistent with a concept of basal ganglia function that postulates a basic, fundamental role in movement sequencing and postural adjustments in anticipation of volitional movements that can neither be replaced nor compensated for once damaged beyond a certain threshold, thus resulting in increasingly degraded motor performance as the degenerative processes progress.

We investigated four different (anatomical) markers of structural disease load and their relationship with brain activity and task performance. Whole brain gray matter as a percentage of total intracranial volume was the marker of structural disease load most often associated with compensation-like changes in these relationships in the rsfMRI analyses. In previous analyses involving many of the same participants, gray matter degeneration accelerates close to disease onset (Tabrizi et al., 2013). However, for both task activation and resting state analyses, significant compensatory and non-compensatory effects were observed using all four measures of structural disease load. In this study, we treated each measure of disease load as statistically independent. Given that the striatum, gray matter and white matter all degenerate, albeit at different rates, during the premanifest stage of HD, they are likely statistically dependent. For future studies, it may prove more useful to integrate all four measures within a multivariate analysis to account for the relationships between the individual measures.

Our definition of compensation was in part derived from the compensation criteria detailed in Cabeza and Dennis Cabeza and Dennis (2013); in particular, that for successful compensation to be present, an increase in activation should be positively associated with an increase in task performance (positive relationship). However, it is possible that there are alternative definitions and underlying mechanisms of compensation that may also be appropriate for future investigation, which are not consistent with our operational definition. For example, compensatory processes may be driven by the downregulation of pathologically high signals or the potential disengagement of brain regions. These mechanisms would be reflected within our model as negative correlations between brain activity (within certain regions) and performance conditional on structural disease load. Furthermore, the current study highlights compensatory activity in regions such as the FFG and the hippocampus which are not routinely associated with general cognitive processing it is this potential recruitment of alternative pathways that we will look to investigate further in future studies with longitudinal data, in addition to the negative correlations and the changes in these associations over time.

We recognize that our study has a number of limitations. Despite a large sample size and our *a priori* definition of a compensation model, we found comparatively little evidence for widespread neural compensation in our presymptomatic HD gene carriers. This may reflect a true negative finding that there is little underlying compensation in the presymptomatic phase of neurodegeneration occurring during the states we chose for fMRI measurement. The tasks we examined (working memory and motor tasks) showed no large difference in behavior between healthy and premanifest HD groups. Such findings are consistent with neural compensation, but are of course also consistent with the possibility that compensation in those particular tasks does not play a large role. Alternatively, it may be a false negative finding; that despite our large sample size, statistical power may be inadequate to detect compensation. In particular, our operational definition of compensation hypothesizes an interaction between different measures in the context of the linear statistical models employed here. Power to detect such interactions is inherently much lower than that required to detect so-called main effects of individual variables. We have argued that such interactions are a necessary, but previously overlooked aspect of the definition of disease compensation. Future work will be able to explicitly investigate whether the compensation effects identified here replicate in the same or different cohorts; and investigate whether a single weighted measure of structural disease load across the brain may be more appropriate than the four measures (necessitating correction for multiple comparisons) used here.

This study has highlighted the complexity of operationally defining compensation in neurodegenerative diseases such as HD. The previous literature in HD has thus far failed to address this issue, instead making assumptions of putative compensation based solely on increased activation or connectivity in high disease load groups compared to those with lower levels of disease or healthy controls and typically did not include performance level (Scheller et al., 2014). We cannot therefore differentiate between functional changes that are due to HD pathology and those that represent compensatory behavior. Here, we explicitly modeled the interaction between fMRI signals and structural disease load as a predictor of cognitive and motor performances taking into account both variability in structural disease load and performance. By explicitly considering the joint relationships between brain function, task performance and structural disease load, we have identified regions in which increased brain activity (or connectivity) in those closer to onset is associated with preserved performance. We contend that relatively preserved performance is an additional necessary component for demonstrations of neuronal compensation. This is a new study of neurodegenerative disease that has explicitly characterized and measured neural compensation using fMRI in combination with structural measures of disease load and markers of task performance. Despite the exploratory nature of our findings, they may have mechanistic implications for the future planned testing of potential disease modifying agents in the presymptomatic phases of neurodegenerative diseases such as HD, where it may be important to monitor for preservation of compensatory activity and connectivity.

## Role of the Funding Source

This work was funded by the CHDI Foundation (TrackOn_RecID_A-4738), the Wellcome Trust (100227) (GR), and the Medical Research Council (MR/L012936/1) (SJT, MP). and supported by the National Institute for Health Research (NIHR) University College London Hospitals (UCLH) Biomedical Research Centre (BRC). The main study sponsor (CHDI_Foundation) contributed to the conception of the study and the study design but was not responsible for data collection, data analysis, data interpretation, or writing of the report. BB, the sponsor's Science Director, also contributed to data interpretation and writing of the report.

## Author Contributions

All authors had full access to all the data in the study and had final responsibility for the decision to submit for publication.

Overall leadership of Track-On HD research project: Sarah Tabrizi.

Conception, organization and execution of the TrackOn-HD research project: Stefan Klöppel, Alexandra Durr, Raymund Roos, Blair Leavitt, Bernhard Landwehrmeyer, Ralf Reilmann, Beth Borowsky, Marina Papoutsi, Hans Johnson, Gail Owen, Julie Stout, Rachael Scahill, Geraint Rees and Sarah Tabrizi.

Analysis of the imaging data: Stefan Klöppel, Sarah Gregory, Elisa Scheller, Lora Minkova, Adeel Razi, Rachael Scahill and Geraint Rees.

Statistical Analyses: Jeffrey Long and James Mills.

Writing of the manuscript: Stefan Klöppel and Sarah Gregory.

All co-authors reviewed and critiqued the manuscript.

Track-On HD Investigators A Coleman, J Decolongon, M Fan, T Koren (University of British Columbia, Vancouver); C Jauffret, D Justo, S Lehericy, K Nigaud, R Valabrègue (ICM and APHP, Pitié- Salpêtrière University Hospital, Paris). A Schoonderbeek, E P ‘t Hart (Leiden University Medical Centre, Leiden); H Crawford, E Johnson, J Read, C Berna, D Hensman Moss (University College London, London); D Craufurd (Manchester University, Manchester); N Weber (George Huntington Institute, Munster); I Labuschagne (Monash University, Melbourne); M Orth (Ulm University, Ulm).

## Conflict of Interest Disclosures

None.

## References

[bb0100] Ashburner J. (2007). A fast diffeomorphic image registration algorithm. NeuroImage.

[bb0015] Barulli D., Stern Y. (2013). Efficiency, capacity, compensation, maintenance, plasticity: emerging concepts in cognitive reserve. Trends Cogn. Sci..

[bb0105] Beckmann C.F., DeLuca M., Devlin J.T., Smith S.M. (2005). Investigations into resting-state connectivity using independent component analysis. Philos. Trans. R. Soc. Lond. B Biol. Sci..

[bb0130] Button K.S., Ioannidis J.P.A., Mokrysz C. (2013). Power failure: why small sample size undermines the reliability of neuroscience. Nat. Rev. Neurosci..

[bb0175] Cabeza R.E., Dennis N.A., Stuss D.T., Knight R.T. (2013). Frontal lobes and aging: deterioration and compensation. Principles of Frontal Lobe Function.

[bb0125] Chambers J.M., Chambers J.M., Hastie T.J. (1992). Data for models. Statistical Models.

[bb0140] Colquhoun D. (2014). An investigation of the false discovery rate and the misinterpretation of p-values. R. Soc. Open Sci..

[bb0035] Georgiou-Karistianis N., Poudel G.R., Domínguez D.J.F. (2013). Functional and connectivity changes during working memory in Huntington's disease: 18 month longitudinal data from the IMAGE-HD study. Brain Cogn..

[bb0095] Glover G.H., Mueller B.A., Turner J.A. (2012). Function biomedical informatics research network recommendations for prospective multicenter functional MRI studies. J. Magn. Reson. Imaging JMRI.

[bb0040] Gray M.A., Egan G.F., Ando A. (2013). Prefrontal activity in Huntington's disease reflects cognitive and neuropsychiatric disturbances: the IMAGE-HD study. Exp. Neurol..

[bb0090] Group Study (1996). Unified Huntington's Disease Rating Scale: reliability and consistency. Huntington Study Group. Mov. Disord. Off. J. Mov. Disord. Soc..

[bb0155] Jenkins B.G., Rosas H.D., Chen Y.C. (1998). ^1^H NMR spectroscopy studies of Huntington's disease: correlations with CAG repeat numbers. Neurology.

[bb0045] Klöppel S., Draganski B., Siebner H.R., Tabrizi S.J., Weiller C., Frackowiak R.S.J. (2009). Functional compensation of motor function in pre-symptomatic Huntington's disease. Brain.

[bb0145] Lambrecq V., Langbour N., Guehl D., Bioulac B., Burbaud P., Rotge J.-Y. (2013). Evolution of brain gray matter loss in Huntington's disease: a meta-analysis. Eur. J. Neurol. Off. J. Eur. Fed. Neurol. Soc..

[bb0115] Li B., Daunizeau J., Stephan K.E., Penny W., Hu D., Friston K. (2011). Generalised filtering and stochastic DCM for fMRI. NeuroImage.

[bb0070] Malejko K., Weydt P., Süßmuth S.D., Grön G., Landwehrmeyer B.G., Abler B. (2014). Prodromal Huntington disease as a model for functional compensation of early neurodegeneration. PLoS One.

[bb0160] Mochel F., Durant B., Meng X. (2012). Early alterations of brain cellular energy homeostasis in Huntington disease models. J. Biol. Chem..

[bb0150] Muhlau M., Gaser C., Wohlschlager A.M. (2007). Striatal gray matter loss in Huntington's disease is leftward biased. Mov. Disord..

[bb0050] Novak M.J.U., Warren J.D., Henley S.M.D., Draganski B., Frackowiak R.S., Tabrizi S.J. (2012). Altered brain mechanisms of emotion processing in pre-manifest Huntington's disease. Brain.

[bb0075] Oldfield R.C. (1971). The assessment and analysis of handedness: the Edinburgh inventory. Neuropsychologia.

[bb0120] Owen A.M., McMillan K.M., Laird A.R., Bullmore E. (2005). N-back working memory paradigm: a meta-analysis of normative functional neuroimaging studies. Hum. Brain Mapp..

[bb0010] Papoutsi M., Labuschagne I., Tabrizi S.J., Stout J.C. (2014). The cognitive burden in Huntington's disease: pathology, phenotype, and mechanisms of compensation. Mov. Disord..

[bb0080] Penney J.B., Vonsattel J.P., MacDonald M.E., Gusella J.F., Myers R.H. (1997). CAG repeat number governs the development rate of pathology in Huntington's disease. Ann. Neurol..

[bb0055] Poudel G.R., Stout J.C., D JFD (2013). Functional changes during working memory in Huntington's disease: 30-month longitudinal data from the IMAGE-HD study. Brain Struct. Funct..

[bb0085] Reilmann R., Bohlen S., Klopstock T. (2010). Grasping premanifest Huntington's disease — shaping new endpoints for new trials. Mov. Disord..

[bb0165] Ross C.A., Tabrizi S.J. (2011). Huntington's disease: from molecular pathogenesis to clinical treatment. Lancet Neurol..

[bb0170] Ross C.A., Aylward E.H., Wild E.J. (2014). Huntington disease: natural history, biomarkers and prospects for therapeutics. Nat. Rev. Neurol..

[bb0060] Scheller E., Abdulkadir A., Peter J., Tabrizi S.J., Frackowiak R.S.J., Klöppel S. (2013). Interregional compensatory mechanisms of motor functioning in progressing preclinical neurodegeneration. NeuroImage.

[bb0180] Scheller E., Minkova L., Leitner M., Klöppel S. (2014). Attempted and successful compensation in preclinical and early manifest neurodegeneration — a review of task FMRI studies. Front. Psychiatry.

[bb0110] Smith S.M., Miller K.L., Moeller S. (2012). Temporally-independent functional modes of spontaneous brain activity. Proc. Natl. Acad. Sci..

[bb0020] Tabrizi S.J., Langbehn D.R., Leavitt B.R. (2009). Biological and clinical manifestations of Huntington's disease in the longitudinal TRACK-HD study: cross-sectional analysis of baseline data. Lancet Neurol..

[bb0005] Tabrizi S.J., Scahill R.I., Durr A. (2011). Biological and clinical changes in premanifest and early stage Huntington's disease in the TRACK-HD study: the 12-month longitudinal analysis. Lancet Neurol..

[bb0025] Tabrizi S.J., Reilmann R., Roos R.A. (2012). Potential endpoints for clinical trials in premanifest and early Huntington's disease in the TRACK-HD study: analysis of 24 month observational data. Lancet Neurol..

[bb0030] Tabrizi S.J., Scahill R.I., Owen G. (2013). Predictors of phenotypic progression and disease onset in premanifest and early-stage Huntington's disease in the TRACK-HD study: analysis of 36-month observational data. Lancet Neurol..

[bb0135] Uttal W.R. (2013). Reliability in Cognitive Neuroscience: A Meta–Meta-Analysis.

[bb0065] Wolf R.C., Vasic N., Schonfeldt-Lecuona C., Landwehrmeyer G.B., Ecker D. (2007). Dorsolateral prefrontal cortex dysfunction in presymptomatic Huntington's disease: evidence from event-related fMRI. Brain.

