# Supplementary material for: Compensation in Preclinical Huntington's Disease: Evidence From the Track-On HD Study
Source: eBioMedicine. 2015 Aug 4;2(10):1420–9. doi: 10.1016/j.ebiom.2015.08.002 (PMC4634199; doi:10.1016/j.ebiom.2015.08.002)
Supplement: Supplementary file 1 — Supplementary material. [file mmc1.pdf]

Supplementary Data

Supplementary Methods

Selection and Description of Participants

HD gene-expansion mutations were initially screened by local diagnostic genetic testing results, which all at-risk and early HD participants had opted to obtain prior to enrolment into the Track-On study (recruitment was limited to individuals who had previously undergone predictive genetic testing resulting in a CAG repeat length ≥ 40). In order to increase the yield of disease-related changes in the premanifest cohort, a burden of pathology selection criterion was used. Burden of pathology was determined by (CAG- 35·5) × age.[1] A threshold of >250 for the burden of pathology score was set, which approximates to ~15 years to estimated disease onset.[2] At recruitment, in addition to a burden of pathology score of > 250, premanifest HD expansion mutation-carriers also required a total motor score of ≤5 in the UHDRS motor assessment [3] indicating lack of significant motor signs. CAG-expansion as well as definitive determination of expansion length was confirmed by analysis of a post-recruitment blood sample sent to ***BioRep***® Technologies Inc (Milan). Repeat length was determined using a PCR assay [4,5] and highly accurate size markers using the MegaBace Fragment Profiler Software (General Electric, Buckinghamshire, UK). When CAG repeat size, measured using this technique, was compared to reported repeat size at recruitment, which relied on historic diagnostic genotyping using various techniques and size markers dependent on the standard operating procedures of the respective diagnostic laboratories, 37% of premanifest subjects were found to have CAG measurements one or two repeats shorter than expected. As a result of centralized resizing of CAG repeats, 13% of the preHD group did not reach the severity threshold for disease burden (>250). Written informed consent was obtained from all subjects, who met the additional inclusion criteria: aged between 18 and 65 years; ability to tolerate MRI and biosample collection; absence of major psychiatric disorder or history of significant head injury at time of enrolment. Subjects were not excluded based on medication usage, unless actively part of an experimental therapeutic trial. In general, comorbid medical conditions were noted, but unless they prevented subject assessment were not considered exclusions.

Measures

Primary assessments were completed using a fully electronic data capture and content management system which provided secure access to the system for sites worldwide. All subject phenotypic data was pseudonymised and securely stored in the European Huntington's Disease Network (EHDN) Clinical Trial Management System (CTMS, Ulm). From each subject, plasma, and white cell buffy coat pellet samples were collected as well as additional samples for RNA and DNA analysis and generation of lymphoblastoid cell lines. Pseudonymised biosamples were stored in a secure biorepository (BioRep, Milan). Control of image acquisition protocols, quality, and equipment specification was centralised under a contract research organization (IXICO Ltd, London), and all images stored in a secure database at the Laboratory of Neuro Imaging, UCLA (LONI, Los Angeles). Stringent quality control and assurance measures were implemented. All clinical staff were trained and assessed for inter-rater reliability prior to the start of the study. All outcome measures were automated or computer-administered as far as possible and the same equipment was used at each site. A centralized database was used to store all data and was monitored online daily. Oversight of the UHDRS clinical scale administration was provided by each clinical site PI.

Biosamples

All subjects provided up to 50ml of blood for biomarker analysis. These samples were collected by the site neurologist and were donated with the understanding that all specimens would be used for HD-related research, and that they would be pseudonymised and stored at a secure central biorepository. Samples were processed on-site without delay to extract good quality plasma and divide it into 500µL aliquots for rapid freezing. All consumables were provided by the commercial repository (BioRep, Milan) on a per-patient basis and shipped to BioRep on a monthly basis. The samples for DNA and lymphoblastoid cell lines were shipped on the day of collection. Plasma samples and PAXgene samples for RNA were collected locally, stored locally at -80C, and shipped on dry ice to BioRep at monthly intervals. DNA and DNA derived from lymphoblastoid cell lines were used to confirm the size of the CAG expansion mutation within the HD gene for all subjects, for research purposes only. In the future these biosamples may be used to identify genetic modifiers of HD, in particular genetic modifiers of age of onset, rate of progression and phenotypic characteristics presentations. For this purpose, one tube of ACD blood was collected for the extraction of DNA, the generation of lymphoblastoid cell lines and the cryopreservation of lymphocytes. Plasma samples were collected in EDTA tubes (3 × 6ml) for proteomic, ELISA and meso-scale analysis, and lithium-heparin tubes (2 × 6ml) for metabolomic analysis. Two PAXgene RNA blood tubes (2.5ml) were collected for the isolation of RNA for microarray or other RNA biomarker analysis.

Cognitive Assessments

The cognitive battery lasted approximately 60 minutes and included a broad range of tests known to be sensitive in HD. The tests were administered to all subjects by paper and pencil in the case of standard clinical neuropsychological tasks, or by using identical model tablet PCs with custom-designed software, and a standardized stylus and mouse input device. A full description of most tasks within the cognitive battery has been described previously.[6] Processing speed was measured using the Stroop Test - word reading condition, in which the participant must read as many words as possible in 45 seconds from a list of the names of colors printed in black ink and the number of words read correctly is the primary variable. The Symbol Digit Modalities Test (SDMT) is a test of visuomotor integration, measuring visual attention and motor speed. The participant is given 90 seconds to match symbols and digits as quickly as possible, following a key located at the top of the page, and the total number of correct responses is recorded. Paced Tapping provides a measure of psychomotor functioning, including timing. The task begins with the repeated presentation of a tone at a constant rate (3Hz). The participant is instructed to begin to tap with alternating thumbs at the same rate as the tone, when the participant feels that they have a sense of the timing. Once the participant begins to tap, the tone continues for another 12 taps, but is then discontinued. The participant will then attempt to maintain the timing of the tap for another 31 taps. This sequence is repeated 4 times for a total of 5 trials. For the Circle Tracing task, the participant traces a 90mm-diameter circle as quickly and accurately as possible, aiming to stay within the 5mm annulus, using a stylus on the horizontally-placed tablet PC. There were three 45-second trials in each of two visual feedback conditions. In the direct condition subjects are able to view their hand and the tablet directly during tracing; in the indirect condition their hand and the tablet are obscured from sight by a suspended cloth and visual feedback is instead presented on a second vertically-placed monitor. The primary variable is the length traced within the annulus boundaries across the 45-second tracing period, which is an indication of visuomotor integration and motor planning. The Map Search Task is a subtest from the Test of Everyday Attention,[7] and measures visuospatial selective attention. For the task, participants were presented with a large

A3 sized map fitted into a clear plastic sleeve. They were timed for two minutes while they searched for, and used a pen to circle, a target symbol that occurred in multiple places on the map among other distracter symbols. The map was visually cluttered, similar to any road map, and displayed a portion of the city of Philadelphia in the United States. The participant began performing the task using a pen of a given color, and then after one minute, the examiner exchanged the pen for a different color to facilitate differentiation of those responses made in the first and second minutes of testing. Test performance was measured as the number of correctly circled target symbols, scored separately at one minute and at two minutes. The maximum possible score was 80. The Cancellation Task measures selective attention. Across several trials (typically 3 90-sec trials), participants must locate particular stimuli or combinations of stimuli (for example small squares with a small line on a given side, or pictures of fruits) randomly distributed among distractors (squares with a line on another side, pictures of other fruits or other similar looking objects) on a tablet computer. Spot the Change is a visual working memory task in which the participant is required to view an array of five colored squares that is presented for 250ms (short enough so that subjects cannot verbally encode the items). After 1000ms a similar array is presented with one of the squares encircled. Subjects decide whether the square within the circle is the same as in the original array or has changed in color. The outcome is Cowan's *K* formula for estimating the number of items encoded at each set size [8], which assesses individual differences in the flexibility of the scope of attention, such that individuals with greater working memory are able to "zoom out" to apprehend and sustain more items from the visual field. The Mental Rotation Task is used to assess the ability to mentally rotate 3-dimensional stimuli. The stimuli consisted of images of cubes, attached to each other by sharing a common side or sides.[9,10] On each trial, a pair of stimuli was presented. One figure in the pair was either a rotation of the other figure, or a rotation of the mirror image of the other figure. Participants responded by indicating whether the rotated figure was identical to ("same") or a mirror image of ("mirror") the comparison figure. The stimuli were presented using six of degrees of rotation across four difficulty levels [10–12]. Testing began with ten practice trials, which were followed by 48 experimental trials comprised of 12 trials each at the four difficulty levels, balanced for "same" and "mirror" conditions. For simplicity, we subsequently refer to the four angles as 5, 65 (e.g., 65 and 305, which are both equal distance from 0), 125 (e.g., 125 and 245, which are both equal distance from 0), and 185 degrees. The outcome measures included accuracy (percent correct), response time (calculated for correctly identified targets only) and speed-accuracy trade-off (correlation between accuracy and response times). Additional tasks not included in Stout and colleagues (2012) were the cancellation task and circle tracing with serial 3s. The cancellation task measures selective attention. Across several trials (typically 3 90-sec trials), participants must locate particular stimuli or combinations of stimuli (for example small squares with a small line on a given side, or pictures of fruits) randomly distributed among distractors (squares with a line on another side, pictures of other fruits or other similar looking objects) on a tablet computer. Circle tracing with serial 3s was added to the existing circle tracing task in which participants perform the circle tracing task while at the same time counting backwards condition (e.g. counting from 100 backwards by 3s). The participant traces a 90mm diameter circle on a horizontal computer tablet while trying to remain within a 5 mm error margin that is indicated by a white annulus on a grey background. The participant attempts the task while indirectly viewing stylus movement on a separate, vertical computer screen with hand and stylus movement occluded from view (3 trials, 45 seconds each). The counting backwards component of this task requires participants to count aloud backwards by some number while performing the Circle Tracing task. Prior to combining circle tracing and counting backwards, participants will practice counting backwards alone.

Global Cognitive Composite

The resting state fMRI data were not associated with a specific task, such as the verbal working memory task and therefore, in the resting state analyses we were testing for effects associated with a more global cognitive network. The global cognitive composite measure, which included those tasks previously shown to be sensitive in this patient population to longitudinal change, thus combined information on several cognitive variables in an efficient manner. Not only was this more appropriate for investigating the global cognitive network, but it potentially provides a better indicator of underlying cognitive performance and increasing variability that can perhaps yield higher statistical power.

The Global Cognitive Composite was based on a principal components analysis using: the modified Stroop word reading test (number of words correct in 45s), SDMT (total number correct in 90 s), Paced Tapping (3Hz, log of the standard deviation of intertap intervals), Circle Tracing, indirect condition (length (cm) of ink laid within annulus), Map Search test (total correctly found in 1 min), Cancellation task (digit number correct in 90s), Spot the Change task (group set size 5, number correct), Mental rotation task (% correct), and Serial subtraction during Circle Tracing indirect condition (total number of correct subtractions). This analysis yielded a single component with an Eigenvector > 1. For the component with eigenvalue > 1, all variables yielded similar values (ranging from ·22 to ·41), making it reasonable to define the Global composite as an average of the standardised scores (multiplied by a constant) where s_{x1} - s_{x9} give the standard deviations for each variable $x_1 – x_9$

$$C3_i = 0.3\left[\frac{x_{1i}}{\sigma_{x_1}} + \frac{x_{2i}}{\sigma_{x_2}} - \frac{x_{3i}}{\sigma_{x_3}} + \frac{x_{4i}}{\sigma_{x_4}} + \frac{x_{5i}}{\sigma_{x_5}} + \frac{x_{6i}}{\sigma_{x_6}} + \frac{x_{7i}}{\sigma_{x_7}} + \frac{x_{8i}}{\sigma_{x_8}} + \frac{x_{9i}}{\sigma_{x_9}}\right]$$

UHDRS '99 Motor

The examination was performed by raters certified by the EHDN UHDRS-TMS online certification (www.euro-hd.net). This requires successful rating of three sample patients, filmed during UHDRS-TMS application, within a range defined as acceptable by experts in the field (as determined by a task force of the EHDN Motor working group).

Quantitative Motor (Q-Motor) Assessments

A pre-calibrated and temperature controlled force transducer (Mini-40, ATI Industrial Automation, NC, USA) was used for all force transducer based Q-Motor assessments.[13] The force transducer had a circular plane contact surface measuring 40 mm in diameter. All data were sampled at 400 Hz, stored and analyzed on a flexible laboratory computer system (WINSC/WINZOOM, University of Umeå, Sweden). All sites were equipped with identical systems and software. All data evaluation was performed blinded in the motor laboratory at the George Huntington Institute (www.ghi-muenster.de) Muenster using automated software. Assessment of involuntary choreatic movements, by means of Grip Force Variability were performed as described previously.[14] We conducted five trials with both a 250g and 500g object using the dominant and non-dominant hand; the measure for 500g with the dominant hand was used in the current study.

Verbal Working Memory (VWM) fMRI task

Participants were given instructions outside of the scanner and practised each condition inside the scanner. If participants achieved at least 70% correct responses in the 1-back condition, they proceeded to the 2-back. If this criterion was not met, they were given the opportunity to complete a second round of training. Instructions and letters were presented in light grey against a black background with font size scaled according to imaging site-specific mirror-projector setup. The three conditions were presented in a blocked design in a pseudo-randomized order. At the beginning of each block, condition-specific instructions in the respective spoken language were presented on the screen for 4 s. There were 6 blocks per condition, each lasting 30s during which ten letters were displayed. All stimuli were presented for 1500ms with a 750ms rest interval. We excluded 11 participants based on low performance during the 1-back condition (d-prime coefficient of < 0), allowing for high performance variability in both 1-back and 2-back conditions.

Sequential Finger Movement (SFM) fMRI task

Tapping sequences were either simple or complex and each was paced by metronome clicks presented via headphones at a rate of either 0·5 or 1·5 Hz, resulting in slow or fast sequences, respectively. In addition to the task condition, an auditory-only rest (baseline) condition was used in which the metronome clicks were presented to the participant but no movement was required. The experimental paradigm consisted of six blocks, each lasting 20s (simple-slow, simple-fast, complex-slow, complex-fast, rest-slow, and rest-fast). Each block type was presented five times in a pseudo-randomised order. Participants were instructed outside the scanner and performed a practice round in the scanner as for the VWM-task. Low performance in four out of five blocks per condition led to exclusion.

Implementation of multi-site imaging protocol

The current study is a follow-up to the previous TRACK-HD study [15] which already included structural imaging. We chose a motor and working memory task which were both found to perform well in previous multi-site applications.[16–18] To implement resting state fMRI and task fMRI, we followed the published guidelines in the preparation phase and employed recommended tools throughout the data acquisition phase.[19] During the preparation phase we ensured to match equipment (button boxes, receiver coils, sound and light levels, etc.) between sites as much as possible. We consulted with MR-physicists to identify an fMRI sequence which could be implemented on both scanner systems. To make the training phase for the fMRI tasks and the whole scanning procedure (ordering of scans, breaks, etc.) as similar as possible, a detailed manual was written to instruct site staff. All site staff participated in a hands-on training session in London. Before the start of the study, a human phantom as used at all sites to ensure identical settings and instructions. Throughout the study, data quality was monitored visually by IXICO. In parallel, QC software was applied to all scans within three working days of acquisition. TCs with the site staff ensured that any upcoming queries could be dealt with in a timely manner.

Quality control procedures for imaging data

Each structural image was checked for quality by ensuring the imaging protocol parameters were consistent and checking for complete brain coverage, wrap, missing data, motion artefact, noise, inhomogeneity, flow, susceptibility, and other artefacts. All scans were rated on quality with regards to the overall image, cortical boundaries (for brain delineation), grey and white contrast of the cortex and the quality of deep grey structures, such as the caudate. Task fMRI volumes with significant artefacts were detected and repaired using tsdiffana and ArtRepair software (http://www.fil.ion.ucl.ac.uk/spm/ext/). Those scans with more than 1.3% variation in global intensity and 1.0 mm per TR scan-to-scan motion were identified as outliers and repaired by interpolation from the nearest unaffected volumes. On average, approximately 3% of all slices for all participants were corrected by this procedure. Following a histogram-based approach for outlier identification, 6 participants from the motor task and 5 from the VWM-task with more than 13% of low quality volumes were excluded from the subsequent analysis. Six SFM and five VWM task datasets were excluded.

Resting state fMRI and some seed-based correlations can be susceptible to motion. Motion can add variance to timeseries, particularly amongst neighbouring voxels and this can lead to spurious correlations.[20,21] Standard procedures such as the inclusion of motion parameters to adjust for absolute head displacement during correlation analyses may not be sufficient to remove the potential motion artefacts that remain due to the prolonged effect (6-10 seconds) of motion. In fact, a recent paper has suggested that that the comprehensive removal of prolonged changes still requires further modelling.[22] Currently, the issue of how best to model and remove motion-induced variance in resting state fMRI is one that has led to considerable debate and controversy. Recent publications have suggested a series of techniques that can be introduced to remove motion-related artefact such as Global Signal Regression (GSR) and scrubbing or censoring, but importantly these present certain disadvantages which may weigh against their implementation. For example, it has been suggested that GSR is the most successful way of removing residual motion artefact that remains after inclusion of motion parameters and white matter and CSF signal.[22] However, inclusion of GSR within resting-state analysis is a longstanding matter of debate. The global signal contains a mixture of both artefactual and "real" signal and while GSR does successfully remove some of the additional motion artefact, it can also remove some of the signal related to neural activity; this is especially of concern given that the global signal correlates most significantly with gray matter signal. Furthermore, GSR can also introduce artefactual autocorrelations[23] and introduce a negative bias on computed correlations due to zero-centering.[24] Given these concerns and on balance, we chose not to implement GSR as a method of motion correction, particularly as DCM do not examine correlations but instead measure effective connectivity.

Data scrubbing or censoring can also be used to remove volumes or spurious correlations that have been impacted by motion artefacts at either the data pre-processing stage or during correlation analyses respectively. Using data scrubbing, however, not only reduces the amount of data available for analysis, but more importantly, introduces a subjective element due to the required implementation of thresholds. Furthermore, removal of volumes can results in a sampling error and variation in degrees of freedom. Therefore, there is still no universally accepted technique for additional removal of residual motion artefact.

We therefore, took into account these concerns in our approach. In addition to the inclusion of motion parameters, our main strategy for guarding against the effects of motion generated artefacts was through the

implementation of stringent quality control procedures on the raw data and at each stage of pre-processing and analysis. Power et al have shown that the most reliable indicator of quality control is prior to preprocessing, as with our study. For the raw data, we performed a standard preliminary visual scan of all volumes to check for gross motion artefacts and signal drop outs. Images were then further examined for motion using the ArtRepair Movie function which identifies any artefacts due to sudden motion, global dip, and scanner environment and so on. It is possible to remove volumes using this software, but given the reasons detailed above we did not do this. Instead, we used tsdiffana to investigate variability in signal intensity both between volume and between slice variability; this is similar to the DVARS quality control measure used by Power et al., This enabled us to identify any participants with abnormally high levels of motion marked by abrupt changes in signal intensity either between volume or between slices. Although we did not investigate framewise displacement (between-volume head movement), Power et al have shown that changes in signal intensity are closely associated with between volume head movement prior to pre-processing. Following this, as standard, we ensured that all pre-processing was performed successfully, in particular realignment, co-registration to the structural image and normalisation. Finally, prior to the experimental seed-based analyses, we performed a comparable GLM-based analysis to investigate the default mode network in each participant. This network is most closely associated with resting-state data and one of the most clearly identifiable. This enabled us to verify any potential problems or peculiarities with seed-based correlation analyses.One rsfMRI dataset was excluded due to motion

Statistical thresholding for image analyses

The main focus of our study was the compensation analyses. Consequently, we were required to perform individual imaging modality analyses most appropriate for identifying the brain activity and connectivity parameters to be included in the compensation analyses. We therefore, used different threshold for main effects and interaction (i.e. compensation) analyses as we also did in previous work (e.g. [25]). For the whole brain task fMRI, a standard $p<0.001$ cluster-defining threshold was used. When identifying the connectivity parameters to be taken forward from the seed-based and DCM analyses we selected a very conservative threshold. As resting state data generally show extensive correlations and activity at an individual level, we wanted to ensure that the selected connectivity parameters represented genuine connectivity. For the seed-correlation analyses, we used a $p<0.05$ FWE correction, which is standard for SPM analyses. However, for the DCM analyses, a $p<0.05$ FDR correction was more suitable. As DCM analyses were performed outside of SPM and therefore not analyzed under Random Field Theory (RFT), an FWE correction would have been too conservative given the between-connection dependencies.

Task fMRI – Main effects analysis

To characterize the main effects of the VWM task, the 2-back condition was contrasted with the attention condition (0-back); to characterize effects of VWM load, the 2-back condition was compared to the 1-back condition. For the motor task, the main effects of speed (fast vs. slow sequence) and complexity (complex vs. simple sequence) were examined. The resulting parameter maps were normalized to standard MNI space and entered into a 1-sample t-test across all participants to characterize task-specific activations at the group level. Groups were compared using a GLM with adjustment for age, gender, education and site. For between-group comparisons, effects are reported at $p<0.05$ FWE corrected with a voxel-extent threshold >50. We previously used a threshold of 50 voxels when investigating motor activity in HD.[25] The typical extent of a resolution

element in the images is 40 voxels (resels of Gaussian random field theory), which is similar to the chosen extent threshold.

Resting State Connectivity Analyses - Region of Interest selection

For the functional connectivity analyses, co-ordinates for the Dorsolateral Prefrontal Cortex (DLPFC) regions were selected according to an independent meta-analysis of visual and verbal working memory tasks[26]; left hemisphere:(-42, 32, 30); right hemisphere:(42, 32, 30). For the motor network, a seed region within the primary motor cortex (M1) was selected, but in the left hemisphere only, given that all participants included were right-handed (-40, -18, 60). For the effective connectivity analyses, the cognitive network, regions included the left DLPFC (-42, 32, 30), right DLPFC (42, 32, 30), left inferior parietal cortex (-34, -48, 38), right inferior parietal cortex (38, -46, 38) and the anterior cingulate cortex (ACC) (-2, 12, 42). For the motor resting state network, the regions included were the left M1 (-40, -18, 60), the left premotor cortex (PMC) (-24, 0, 54) the right PMC (26, -6, 52), the pre supplementary motor area (SMA) (0, 6, 54), the caudal SMA (-6, -10, 54) the left superior parietal cortex (-22, -68, 58), and the right superior parietal cortex (22, -66, 60). For the seed-based analyses, we used a small 4mm radius for our VOI extraction to ensure that we extracted the principal eigenvariate timeseries which represented the large majority of variance within that region and therefore a timeseries that was strongly representative of that region. For the DCM, we used a standard size 8mm radius as it was not possible to extract a suitable time series for all regions in the model for all participants using a 4mm radius volume of interest. To ensure that the timeseries was extracted from the correct anatomical areas, we used standard anatomical masks to constrain the local peak maxima search. All timeseries used in both the seed-based and DCM timeseries were examined to ensure that they represented an acceptable percentage of the variability of the signal and were, therefore, good representation of the signal in each region. Non-neuronal time-series for both sets of resting-state analyses were extracted from a single voxel located within the pons (white matter signal) (MNI-space coordinates: x=0, y= -24, z= -33) and lateral ventricle (CSF signal) (-1, 45, 3) respectively.

Supplementary Results

Cognitive Network

VWM task – behavioral

Repeated measures ANOVA with VWM load as the within-subject factor and group (controls vs preHD) as between-subject factor showed a significant main effect of VWM load ($F=5{\cdot}511$, $p={\cdot}02$, $\eta^2={\cdot}03$) on task performance (d-prime). No other effects, including interactions between VWM load and group, were significant. The analogous analysis with reaction times as outcome variables yielded no significant main or interaction effects of the factors group or VWM load (Table S1).

VWM task – activation

Performing the VWM task (versus baseline) was associated with substantial BOLD signal increases in the well-established frontoparietal working memory network[26], basal ganglia and thalamus (Table S2; Figure S1) for both controls and preHD participants. There was increased activation in the upper inferior posterior right cerebellum

in controls versus preHD when comparing the 2-back condition to the fixation baseline ($P<0·05$,FWE-corrected) but no differences were detected in cortical structures at this statistical threshold.

VWM task – compensation

Left-handed participants were included in the VWM analyses to ensure a sufficient number of participants for the compensation analyses. While handedness clearly affects the motor system we found no such indication for the working memory system either in the literature or in our data. To fully exclude a possible effect, we performed a re-analyses based on the reviewer's suggestion after excluding all left-handed individuals. We observed a relatively unaltered interaction (i.e. compensation) effect in the right parietal cortex. The original compensation interaction was statistically significant for the right superior parietal cortex (x=39, y=-60, z=45; t= 3·47, p<0·002) and the inferior parietal cortex (x=38, y=-54, z=29; t= 4·18, p<0·001 Reanalysis showed similar effects in the superior parietal cortex (x=39·.4; y=-59; z=45·4, t=2·68, p=0·0093) with the putamen as a measure of disease load and in the inferior parietal cortex using caudate as a measure of disease load (x=37·3; y=-54; z=28, t=2·58 p<0·0012).

Resting state fMRI – effective connectivity and compensation

We did not find any DCM connectivity parameters that were correlated with global cognitive performance and changed with disease load in a fashion predicted by our compensation hypothesis (Table S5). However, we did identify coupling whose relationship to global cognitive performance significantly decreased as structural disease load increased. We found significant decreases in bidirectional connectivity between the left DLPFC and the ACC (lDLPFC to ACC: p=0·037; ACC to lDLPFC: p=0·025) as disease load increased when predicting global cognitive performance (Figure S4; Table S5). This decrease was observed using white matter as a measure of structural disease load, but these results did not survive Bonferroni-correction. The white matter finding, however, is again suggestive of a (non-significant) non-compensatory effect, according to our definition of compensation, in connections from the left hemisphere as preHD expansion mutation-carriers approach onset.

Motor Network

SFM task – behavioral

For the SFM task, differences in performance were assessed using the standard deviation of timing accuracy, defined by the time between a button press and closest click. We employed a 2x2x2 repeated measures ANCOVA, with complexity and speed as within-subject factors and group (controls and preHD) as a between-subject factor, as well as adjusting for age, gender, site and education. The repeated measures ANCOVA showed a significant main effect of complexity ($F=5·385$, $p=0·022$, $\eta^2=0·03$), while all other effects, including interactions with group, did not reach significance (all $P>0·05$). The only significant difference in performance between controls and preHD was for the timing inaccuracy during the simple slow condition (Table S6).

SFM task - activations

Performing a SFM task in the scanner was associated with highly significant activations (versus baseline) of key areas of the motor system including M1 and SMA, as well as dorsal PMC and parietal cortex (Figure S2; Table S7) in both the preHD and control participants. Activations in motor executive regions such as caudal SMA and M1 further increased with faster movements, while performing a more demanding sequence with several

changes in direction resulted in stronger activation of the anterior parts of the SMA. No significant cortical activation differences were observed between preHD and controls.

SFM task – compensation analyses

We employed a similar analytic approach to that described above for the working memory task. However, the compensation model did not reveal any significant relationship between speed and complexity (as performance markers), brain activity and structural disease load consistent with either compensation or non-compensatory changes. This remained the case even when using an exploratory threshold of $p<0{\cdot}01$ uncorrected.

Resting state fMRI – effective connectivity and compensation

We investigated effective connectivity in the anatomically pre-defined motor network using DCM applied to the rsfMRI data. As with the analyses described above, only those connectivity parameters that were significant across all participants at an FDR-corrected threshold of $p<0{\cdot}05$ were included in our compensation model. In predicting variability within grip force performance, we identified significant decreases in several connections as structural disease load increased: right PMC to the preSMA ($p=0{\cdot}043$), preSMA to the right PMC ($p=0{\cdot}044$) right PMC to left PMC ($p=0{\cdot}033$) and from central SMA to left PMC (0·049) (Table S8). These results were observed using white matter volume, grey matter volume and putamen, respectively, as measures of structural disease load, but did not survive Bonferroni-correction. These findings are indicative of a non-compensatory effect according to our definition in both hemispheres in those premanifest HD expansion mutation-carriers close to onset. There were no significant relationships using TMS as a measure of motor performance.

Supplementary Figures

Figure S1: Working memory activations

Increased activations with the working memory task (2-back vs. 0-back and 2-back vs. 1-back) in healthy controls and preHD. Results shown at p<0·05 after FWE-correction for multiple comparisons overlaid on the normalized structural scan from all participants. Colors indicate T- scores.

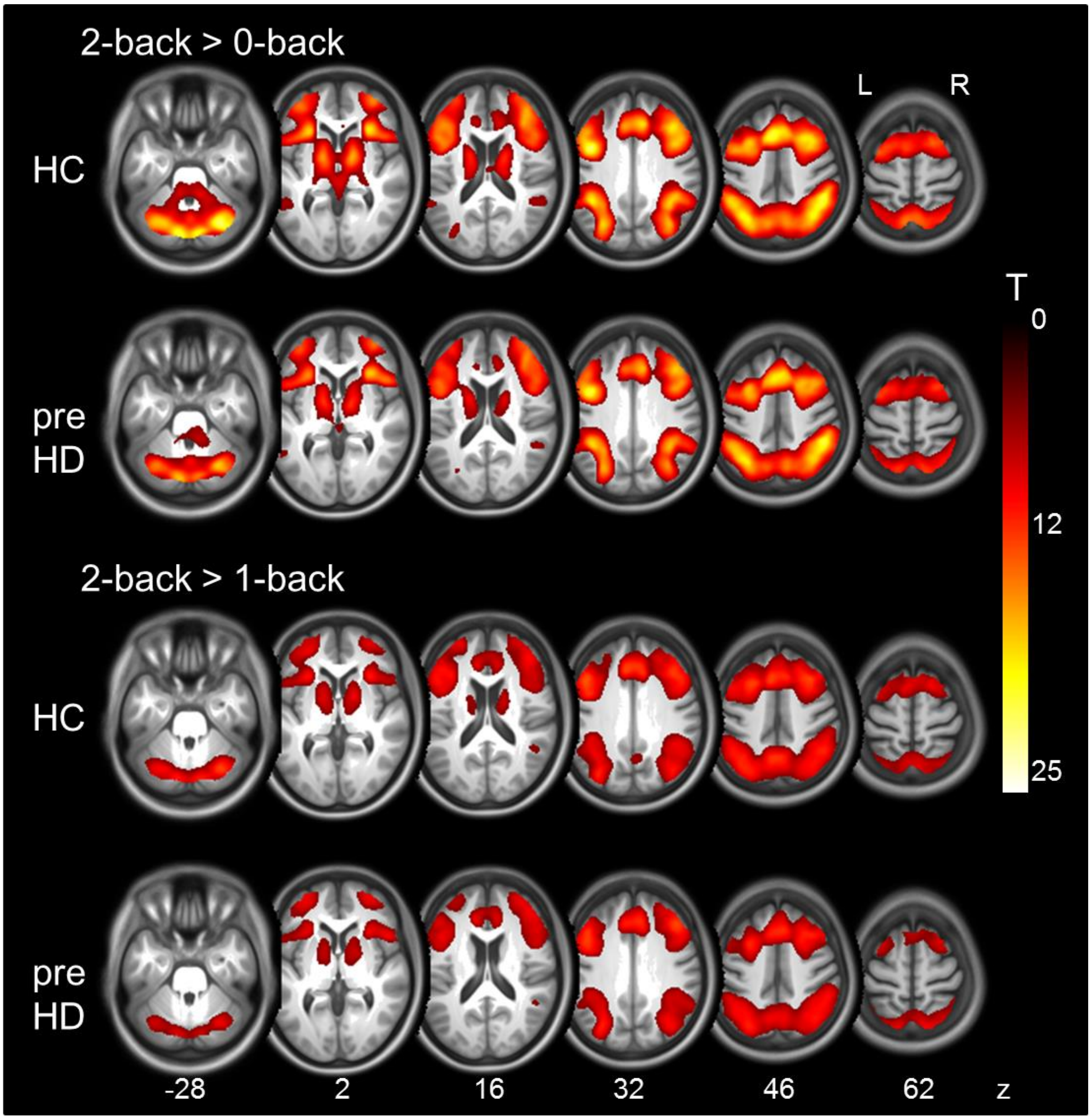

Figure S2: Sequential finger tapping activations

Top row: Increasing activation when complex movements (4-2-3-1) are compared to simple sequences (1-2-3-4). Bottom row: Fast movements compared to slow movements. Results shown at p<0·05 after FWE-correction for multiple comparisons overlaid on the normalized structural scan from all participants. Z-value of MNI-coordinates are provided below. Colors indicate T-score.

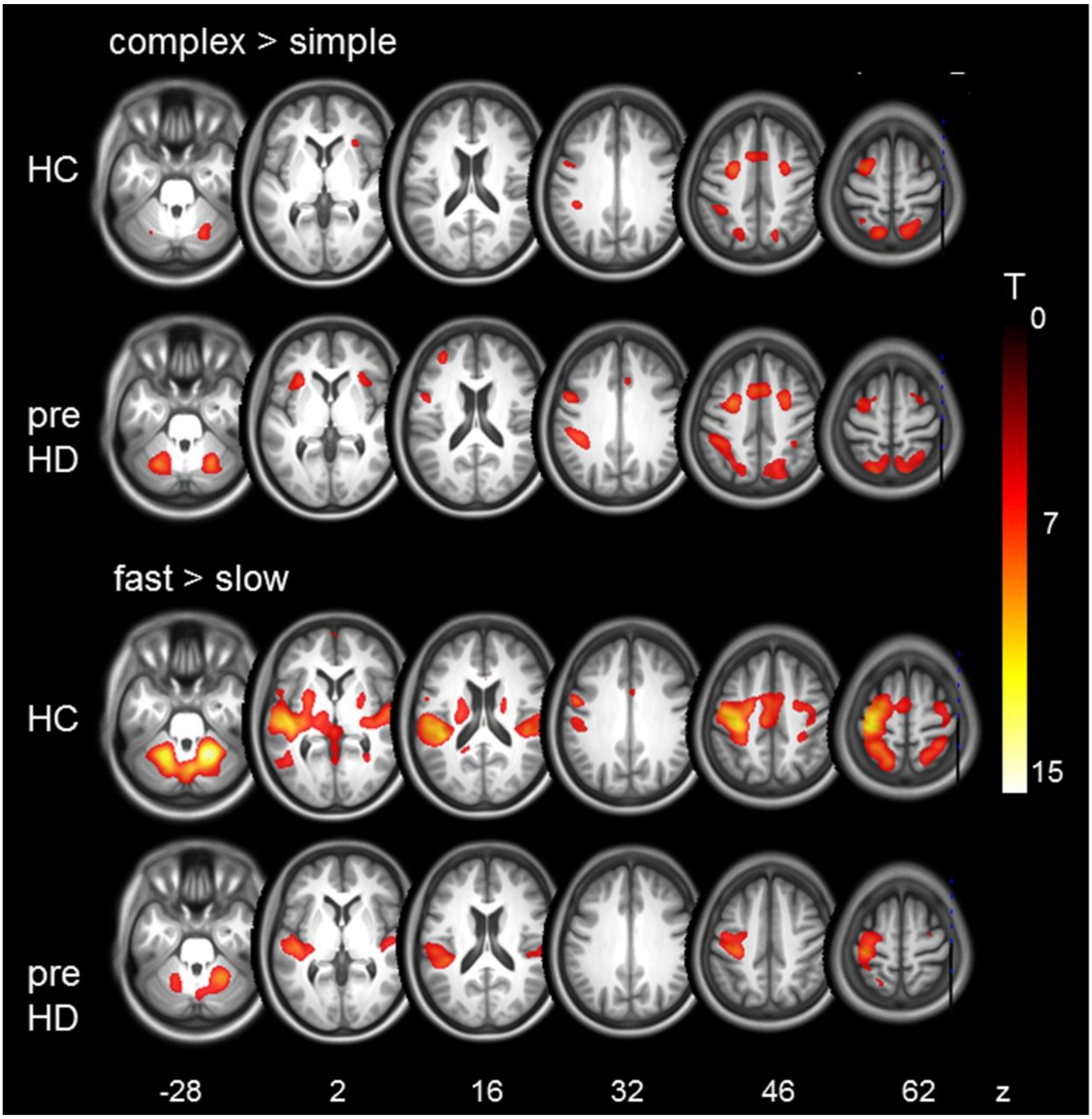

Figure S3: Global cognitive performance as a function of rsfMRI functional connectivity between right DLPFC and the left fusiform gyrus, conditional on grey matter volume as a measure of structural disease load.

For each plot, the upper panel depicts the overlapping ranges of grey matter volume that determine which subsample is selected from the data set that is used to construct each scatterplot. A linear regression line was fit for each scatterplot to aid interpretation.

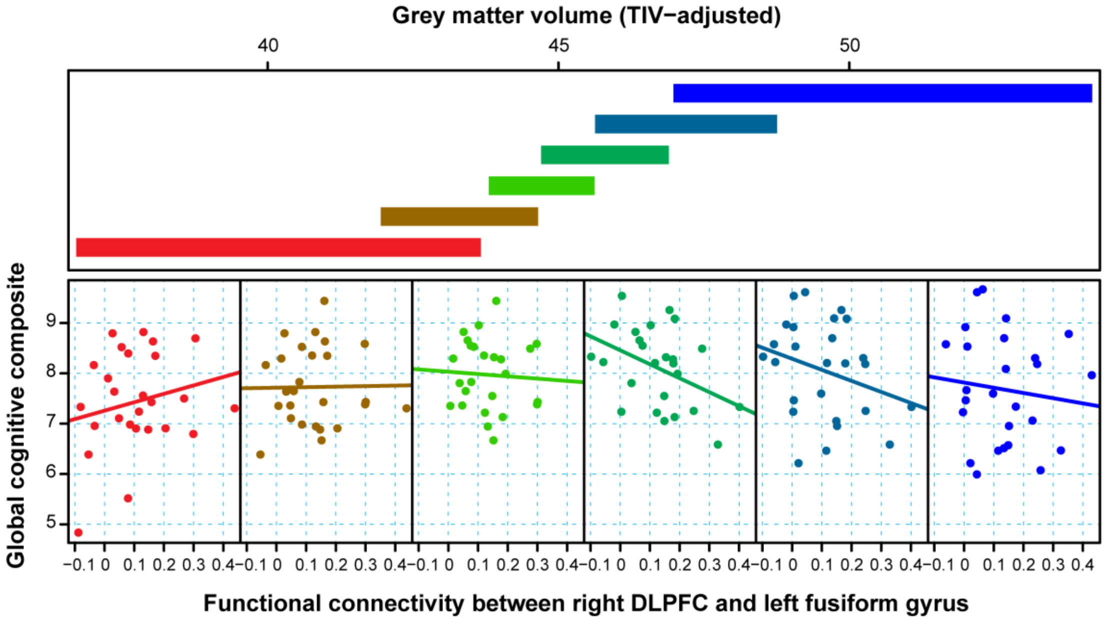

Figure S4: Global cognitive performance as a function of rsfMRI effective connectivity a) from left DLPFC to anterior cingulate cortex and b) from anterior cingulate cortex to left DLPFC, conditional on grey matter volume as a measure of structural disease load.

For each plot, the upper panel depicts the overlapping ranges of grey matter volume that determine which subsample is selected from the data set that is used to construct each scatterplot. A linear regression line was fit for each scatterplot to aid interpretation.

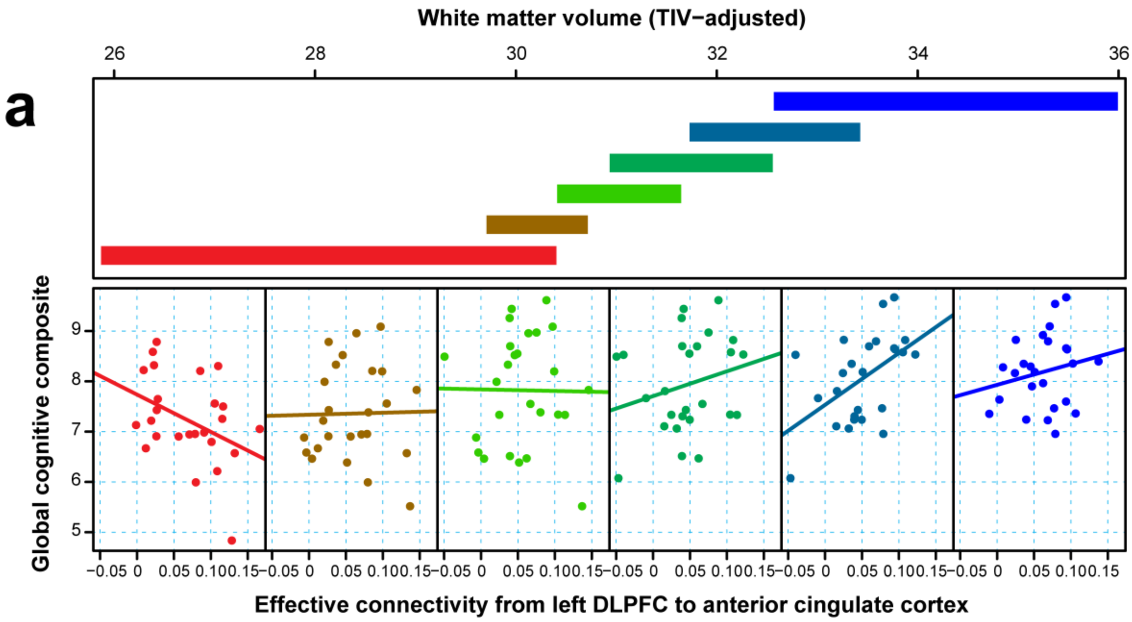

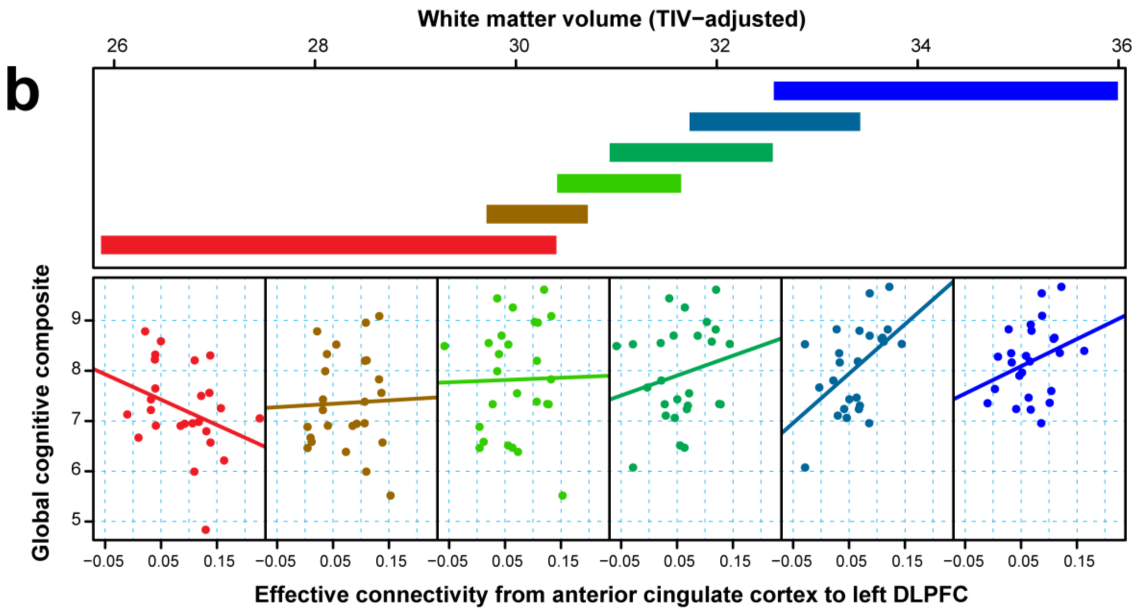

Table S1: Behavioral data verbal working memory task (mean and standard deviation)

HC=Healthy Controls; preHD=premanifest HD; CPO=Cumulative Probability to Onset; DBS=Disease Burden Score

	HC (N = 90)	preHD (N = 89)	Sig. (2-tailed)
**Age (years)**	47·27 *(±10·49)*	42·75 *(±9·33)*	<0·001**
**Gender (female : male)**	53 : 38	37 : 53	>0·05
**Education (years)**	4.01 *(±1·02)*	4·07 *(±1·05)*	>0·05
**UHDRS motor score**	1·23 *(±1·68)*	5·42 *(±4·19)*	<0·001**
**CPO**	n/a	·22 *(±·16)*	n.a.
**DBS**	n/a	302·40 *(±51.56)*	n.a.
**D-prime 1-back**	4·00 *(±·68)*	3·54 *(±·95)*	>0·05
**D-prime 2-back**	2·59 *(±1·03)*	2·26 *(±1·12)*	>0·05
**reaction time 1-back (ms)**	795 *(±181)*	847 *(±195)*	>0·05
**reaction time 2-back (ms)**	945 *(±219)*	1001 *(±230)*	>0·05

Table S2: Main effect of Verbal Working Memory task fMRI

The main effect of the task was calculated across controls and premanifest HD. Local maxima for five regions of interest, drawn from 2-back > 0 back contrast. Threshold at p = 0·05 FWE corrected, k = 50 voxels cluster extent. These maxima are subsequently used to define peaks for rsfMRI-based DCM time series extraction. Abbreviations: IPC: inferior parietal cortex; ACC: anterior cingulate cortex; DLPFC: dorsolateral prefrontal cortex

Region	Hemisphere	MNI coordinates			T
		X	Y	Z	
**inferior parietal cortex**	L	-40·5	-46·5	37·5	31·43
**inferior parietal cortex**	R	37·5	-49·5	39	31·58
**anterior cingulate cortex**	L	-4·5	19·5	42	29·27
**dorsolateral prefrontal cortex**	L	-40·5	25·5	22·5	23·67
**dorsolateral prefrontal cortex**	R	42	25·5	27	26·24

Table S3: Functional connectivity and compensation analyses for the seed region in the right DLPFC.

Connectivity parameters from regions entering compensation analyses, FWE-corrected P<0·05 (left side of table). Significant regions are reported with MNI coordinates and extent threshold (k). Right side of table displays regional interaction tests between the identified functional connections and structural disease load. Columns separate caudate, putamen, grey and white matter (corrected for ICV). Significant at the 0·05 level without (*) or with Bonferroni correction (**).

		**Functional Connectivity**						**Compensation Analyses**							
		**MNI coordinates**						**Caudate**		**Putamen**		**Grey Matter**		**White Matter**	
**Region**	**Hemi**	**X**	**Y**	**Z**	**K**	**Z-score**	**p-value**	**Co-eff**	**p-value**	**Co-eff**	**p-value**	**Co-eff**	**p-value**	**Co-eff**	**p-value**
**Thalamus**	L	-11	2	3	493	5·2	0·001	-0·593	0·592	-0·257	0·762	0·325	0·783	1·836	0·324
**Lateral Occipital Cortex**	L	-17	-67	49	297	5·04	0·002	-0·14	0·891	-0·031	0·968	0·409	0·711	1·485	0·41
**Hippocampus**	L	-20	-30	-5	368	4·91	0·001	-2·044	0·133	-0·457	0·671	-3·239	0·034*	-4·218	0·111
**Postcentral Gyrus**	L	-26	-39	51	117	4·67	0·043	-1·592	0·261	-0·56	0·568	2·497	0·058	1·005	0·692
**Fusiform Gyrus**	L	-30	-75	-15	159	4·87	0·019	-0·838	0·421	-0·902	0·371	-3·006	0·01**	-3·398	0·139
**Middle Frontal Gyrus**	L	-35	35	27	200	4·8	0·009	0·363	0·652	0·867	0·178	0·137	0·873	0·633	0·635
**Insula**	L	38	0	-2	382	5·67	0·001	-0·329	0·75	-0·847	0·314	-0.541	0·633	1·143	0·504
**Middle Temporal Gyrus**	L	-48	-76	6	385	4·96	0·001	-1·054	0·319	-0·201	0·763	-1·279	0·17	-0·105	0·949
**Superior Temporal Pole**	L	-54	3	-3	316	5·11	0·001	-0·408	0·535	-0·417	0·391	-0·32	0·655	-0·212	0·865
**Precuneus**	B	-8	-54	64	1974	5·04	0·001	0·529	0·457	0·385	0·355	0·53	0·426	1·789	0·147
**Cingulate Cortex**	B	0	-30	24	188	4·55	0·011	-0·391	0·524	-0·09	0·859	-0·43	0·596	-0·763	0·542
**Middle Frontal Gyrus**	R	45	33	24	4829	5·5	0·001	-0·32	0·593	0·149	0·765	-0·432	0·506	-1·657	0·12
**Supramarginal Gyrus**	R	57	-43	28	2070	5·34	0·001	0·396	0·64	0·691	0·345	0·077	0·936	-2·229	0·144
**Lingual Gyrus**	L	-11	70	12	4463	5·12	0·001	-0·31	0·736	-0·049	0·944	-1·362	0·127	1·785	0·323
**Inferior Frontal Gyrus**	L	-39	29	13	140	4·48	0·027	-0·633	0·58	-0·542	0·482	-3·193	0·019*	-0·968	0·567
**Superior Temporal Gyrus**	L	-63	-43	6	650	4·92	0·001	-0·997	0·367	-1·537	0·049*	-1·608	0·127	-0·221	0·898
**Anterior Cingulate Cortex**	L	-5	12	39	108	4·35	0·052	-1·724	0·101	-1·621	0·051*	-0·527	0·585	-0·393	0·01
**Superior Temporal Gyrus**	L	-45	-12	-9	248	4·54	0·004	-0·112	0·912	0·259	0·725	-0·919	0·4	-3·074	0·189
**Hippocampus**	R	29	-18	8	107	4·77	0·053	-0·817	0·64	-0·856	0·531	-1·616	0·393	3·115	0·254

Table S4: Functional connectivity and compensation analyses for the seed region in the left DLPFC.

Connectivity parameters from regions entering compensation analyses, FWE-corrected P<0·05 (left side of table). Significant regions are reported with MNI coordinates and extent threshold (k). Right side of table displays regional interaction tests between the identified functional connections and structural disease load. Columns separate caudate, putamen, grey and white matter (corrected for ICV). Significant at the ·05 level without (*) or with Bonferroni correction (**).

		Functional Connectivity						Compensation Analyses							
		MNI coordinates						Caudate		Putamen		Grey Matter		White Matter	
**Region**	**Hemi**	**X**	**Y**	**Z**	**K**	**Z-score**	**p-value**	**Co-eff**	**p-value**	**Co-eff**	**p-value**	**Co-eff**	**p-value**	**Co-eff**	**p-value**
**Thalamus**	L	-3	-4	7	223	4·34	0·004	0·062	0·924	0·051	0·926	1·144	0·19	-0·695	0·561
**Putamen**	L	-33	0	3	219	5·18	0·004	0·419	0·777	-0·023	0·853	3·945	0·01*	2·651	0·249
**Middle Frontal Gyrus**	L	-44	30	27	1677	6·27	0·001	0·596	0·468	0·483	0·474	1·336	0·062	0·805	0·415
**Inferior Parietal Cortex**	L	-45	-39	45	110	4·33	0·039	0·784	0·259	0·797	0·189	1·813	0·007**	3·021	0·025*
**Precuneus**	L	-6	-60	51	103	4·27	0·046	0·194	0·769	0·639	0·943	1·136	0·156	2·113	0·128
**Anterior Cingulate Cortex**	L	-8	17	40	741	5·12	0·001	1·066	0·424	1·203	0·224	3·623	0·004**	3·218	0·119
**Middle Frontal Gyrus**	R	29	47	10	1066	4·76	0·001	0	1	-0·405	0·502	-0·072	0·937	1·031	0·52
**Lateral Occipital Cortex**	R	51	-69	-5	162	4·93	0·013	0·333	0·722	0·49	0·532	-0·284	0·791	-0·287	0·841
**Supramarginal Gyrus**	L	-47	-22	33	389	4·55	0·001	0·893	0·396	0·321	0·69	3·062	0·.003**	1·965	0·249
**Supramarginal Gyrus**	R	60	-25	233	229	4·34	0·001	0·306	0·776	0·537	0·48	1·901	0·042*	1·899	0·303

Table S5: Effective connectivity and compensation analyses: Cognitive Network

Connections entering compensation analyses, FDR-corrected P<0·05 (left side of table). Right side of table displays regional interaction tests between the identified effective connections and structural disease load. Columns separate caudate, putamen, grey and white matter (corrected for ICV). Significant at the 05 level without (*) or with Bonferroni correction (**).Abbreviations: ACC: anterior cingulate cortex; DLPFC: dorsolateral prefrontal cortex; PPC: posterior parietal cortex

	**Compensation Analyses**							
	**Caudate**		**Putamen**		**Grey Matter**		**White Matter**	
**Connection**	**Co-eff**	**p-value**	**Co-eff**	**p-value**	**Co-eff**	**p-value**	**Co-eff**	**p-value**
**l.PPC to r.PPC**	-14·31	0·603	1·864	0·933	0·217	0·743	1·769	0·1
**l.PPC to ACC**	-1·81	0·586	2·389	0·333	-0·244	0·934	-6·122	0·292
**ACC to l.PPC**	-2·603	0·373	1·02	0·633	-0·671	0·812	-4·193	0·433
**l.DLPFC to ACC**	3·759	0·283	1·747	0·496	7·138	0·041*	11·87	0·037*
**ACC to l.DLPFC**	2·382	0·47	1·051	0·669	5·411	0·09	11·14	0·025*
**l.DLPFC to r.DLPFC**	1·099	0·675	0·167	0·934	-3·406	0·178	0·865	0·866
**r.PPC to l.PPC**	-21·17	0·431	-12·75	0·542	-0·116	0·857	2·083	0·075
**r.PPC to ACC**	-3·202	0·302	0·73	0·761	-0·949	0·738	-3·56	0·501
**ACC to r.PPC**	-2·871	0·299	1·072	0·617	0·094	0·972	-1·094	0·812
**r.PPC to l.DLPFC**	1·79	0·521	0·279	0·888	3·634	0·098	7·207	0·157
**r.DLPFC to r.PPC**	1·812	0·537	0·949	0·676	3·978	0·091	7·935	0·134
**r.DLPFC to l.DLPFC**	0·677	0·788	-0·11	0·955	-3·446	0·198	1·362	0·774
**r.DLPFC to ACC**	-4·461	0·237	-3·788	0·171	-3·22	0·422	3·419	0·647
**ACC to r.DLPFC**	-3·642	0·289	-3·23	0·202	-3·191	0·392	1·904	0·772

Table S6: Behavioral data SFM task (mean and standard deviation)

HC=Healthy Controls; preHD=premanifest HD; CPO=Cumulative Probability to Onset; DBS=Disease Burden Score

	HC (N = 82)	preHD (N = 74)	Sig. (2-tailed)
**Age (years)**	·	41·16<br>*(8·66)*	<0·001**
**Gender (female : male)**	50:32	35:39	0·088
**Education (years)**	4·11<br>*(0·98)*	4·11<br>*(0·99)*	0·992
**CPO**	n/a	0·01<br>*(0·116)*	-
**DBS**	n/a	295·51<br>*(48·63)*	-
**Cue-response interval, SD, ms (simple slow)**	137·42<br>*(57·84)*	156·50<br>*(51·43)*	0·040*
**Cue-response interval, SD, ms (simple fast)**	85·40<br>*(42·43)*	91·09<br>*(38·98)*	0·192
**Cue-response interval, SD, ms (complex slow)**	164·53<br>*(77·66)*	168·80<br>*(51·63)*	0·216
**Cue-response interval, SD, ms (complex fast)**	90·88<br>*(42·95)*	99·47<br>*(36·32)*	0·072

Table S7: Main effect of the SFM task.

The main effect of the task was calculated across controls and premanifest gene carriers. This resulted in activations of left primary motor cortex (lM1), left pre-SMA (pSMA), left caudal SMA (cSMA), bilateral dorsal premotor cortex (lPMd, rPMd) and bilateral superior parietal cortex (lSPC, rSPC). Increased complexity of sequential movements resulted in stronger activations in the pSMA, bilateral PMd and bilateral SPC, while increased speed of sequential movements lead to stronger activation in cSMA and lM1 areas.

Region	Hemisphere	MNI coordinates			F	Z
		X	Y	Z		
**Main effect: COMPLEXITY**						
	L	-6	10	46	65·64	7·80
**Dorsal premotor cortex (lPMd)**	L	-25	-3	48	121·33	>8·00
**Dorsal premotor cortex (rPMd)**	R	25	-1	48	82·94	>8·00
**Superior parietal cortex (lSPC)**	L	-15	-70	57	117·34	>8·00
**Superior parietal cortex (rSPC)**	R	16	-73	54	143·14	>8·00
**Main effect: SPEED**						
**Caudal SMA (cSMA)**	L	-4	-4	52	38·62	6·01
**Primary motor cortex (lM1)**	L	-32	-28	54	140·14	>8·00
**Dorsal premotor cortex (lPMd)**	L	-28	-16	60	71·48	>8·00
**Dorsal premotor cortex (rPMd)**	R	27	-12	64	35·02	5·72
**Superior parietal cortex (lSPC)**	L	-21	-64	-51	34·17	5·65
**Superior parietal cortex (rSPC)**	R	24	-52	62	28·20	5·12

Table S8: Effective connectivity and compensation analyses: Motor Network

Connections entering compensation analyses, FDR-corrected P<0·05 (left side of table). Right side of table displays regional interaction tests between the identified effective connections and structural disease load. Columns separate caudate, putamen, grey and white matter (corrected for ICV). Significant at the ·05 level without (*) or with Bonferroni correction (**). Abbreviations: cSMA: caudal supplementary area; pSMA: pre-supplementary area; PMC: premotor cortex; PPC: posterior parietal cortex.

		**Compensation Analyses**							
		**Caudate**		**Putamen**		**Grey Matter**		**White Matter**	
	**Connection**	**Co-eff**	**p-value**	**Co-eff**	**p-value**	**Co-eff**	**p-value**	**Co-eff**	**p-value**
**Total Motor Score**	**pSMA to l.PMC**	-3·173	0·377	1·475	0·593	-2·628	0·514	-4·485	0·492
	**cSMA to l.PMC**	-6·114	0·137	-2·057	0·484	-2·351	0·489	3·712	0·609
	**l.PMC to pSMA**	-4·225	0·212	0·58	0·825	1·67	0·673	-2·788	0·654
	**l.PMC to cSMA**	-5·582	0·098	-0·496	0·838	-2·26	0·411	1·364	0·806
	**l.PMC to r.PMC**	-5·889	0·094	-1·575	0·529	-4·22	0·097	3·824	0·525
	**l.PPC to r.PPC**	-2·252	0·158	-0·903	0·28	-0·285	0·845	-0·001	>0·999
	**cSMA to pSMA**	-3·762	0·156	-1·502	0·548	1·346	0·655	-2·863	0·578
	**pSMA to cSMA**	-3·466	0·182	0·11	0·964	0·064	0·983	-4·428	0·381
	**pSMA to r.PMC**	-3·768	0·368	3·165	0·331	-2·356	0·577	-3·967	0·525
	**cSMA to r.PMC**	-2·715	0·534	3·942	0·294	3·184	0·463	2·078	0·784
	**r.PMC to pSMA**	-4·688	0·251	1·841	0·554	-2·21	0·608	-3·118	0·6
	**r.PMC to cSMA**	-2·776	0·488	4·633	0·133	1·159	0·751	2·227	0·747
	**r.PMC to l.PMC**	-2·65	0·221	-0·474	0·785	-4·006	0·066	-1·559	0·707
	**r.PPC to l.PPC**	-2·803	0·113	-1·443	0·253	-0·342	0·813	-0·51	0·868
	**r.PMC to l.PPC**	-5·63	0·136	-1·637	0·531	-5·065	0·065	3·602	0·575
**Grip Force Variability**	**pSMA to l.PMC**	-2·905	0·176	-2·016	0·397	-2·224	0·188	-3·415	0·378
	**cSMA to l.PMC**	-4·384	0·075	-3·508	0·049*	-2·629	0·191	-1·192	0·783
	**l.PMC to pSMA**	-2·614	0·197	-2·587	0·105	-3·257	0·161	-3·348	0·349
	**l.PMC to cSMA**	-2·907	0·151	-1·968	0·181	-2·627	0·104	-1·162	0·724
	**l.PMC to r.PMC**	-1·359	0·52	-1·626	0·281	-2·255	0·131	-0·914	0·797
	**l.PPC to r.PPC**	-0·676	0·477	-0·493	0·475	-0·399	0·64	-2·916	0·077
	**cSMA to pSMA**	-0·515	0·746	-1·56	0·302	-0·042	0·981	-4·357	0·149
	**pSMA to cSMA**	-0·17	0·913	-0·636	0·664	-0·047	0·978	-4·804	0·165
	**pSMA to r.PMC**	-0·803	0·747	-1·118	0·57	-2·296	0·35	-7·285	0·044*
	**cSMA to r.PMC**	-0·865	0·739	0·639	0·78	0·828	0.745	-7·954	0·073
	**r.PMC to pSMA**	-1·04	0·669	-1·707	0·363	-2·694	0·.286	-7·003	0·043*
	**r.PMC to cSMA**	-1·198	0·616	-0·085	0·964	-2·005	0·348	-6·38	0·114
	**r.PMC to l.PMC**	0·046	0·972	-0·089	0·935	-2·791	0·033*	-4·226	0·088
	**r.PPC to l.PPC**	0·123	0·908	-0·273	0·722	-0·308	0·716	-3·005	0·092
	**r.PMC to l.PPC**	1·57	0·489	-1·736	0·271	-2·005	0·348	-0·732	0·847

Supplemental References

1 Penney JB, Vonsattel JP, MacDonald ME, Gusella JF, Myers RH. CAG repeat number governs the development rate of pathology in Huntington's disease. *Ann Neurol* 1997; **41**: 689–92.

2 Langbehn DR, Brinkman RR, Falush D, Paulsen JS, Hayden MR. A new model for prediction of the age of onset and penetrance for Huntington's disease based on CAG length. *Clin Genet* 2004; **65**: 267–77.

3 Huntington Study Group. Unified Huntington's Disease Rating Scale: reliability and consistency. Huntington Study Group. *Mov Disord Off J Mov Disord Soc* 1996; **11**: 136–42.

4 Riess O, Noerremoelle A, Soerensen SA, Epplen JT. Improved PCR conditions for the stretch of (CAG) n repeats causing Huntington's disease. *Hum Mol Genet* 1993; **2**: 637–637.

5 Warner JP, Barron LH, Brock DJH. A new polymerase chain reaction (PCR) assay for the trinucleotide repeat that is unstable and expanded on Huntington's disease chromosomes. *Mol Cell Probes* 1993; **7**: 235–9.

6 Stout JC, Jones R, Labuschagne I, *et al.* Evaluation of longitudinal 12 and 24 month cognitive outcomes in premanifest and early Huntington's disease. *J Neurol Neurosurg Psychiatry* 2012; **83**: 687–94.

7 Robertson IH, Ward T, Ridgeway V, Nimmo-Smith I. The Test of Everyday Attention. Bury St. Edmunds: Thames Valley Test Company, 1994.

8 Cowan N. The magical number 4 in short-term memory: a reconsideration of mental storage capacity. *Behav Brain Sci* 2001; **24**: 87–114; discussion 114–85.

9 Peters M, Battista C. Applications of mental rotation figures of the Shepard and Metzler type and description of a mental rotation stimulus library. *Brain Cogn* 2008; **66**: 260–4.

10 Shepard R, Metzler J. Mental rotation of three dimensional objects. *Science* 1971; : 701–3.

11 Bethel-Fox CE, Shepard R. Mental rotation: effects of stimulus complexity and familiarity. *J Exp Psychol Hum Percept Perform* 1988; : 12–23.

12 Lineweaver TT, Salmon DP, Bondi MW, Corey-Bloom J. Differential effects of Alzheimer's disease and Huntington's disease on the performance of mental rotation. *J Int Neuropsychol Soc* 2005; **11**: 30–9.

13 Reilmann R, Kirsten F, Quinn L, Henningsen H, Marder K, Gordon AM. Objective assessment of progression in Huntington's disease: A 3-year follow-up study. *Neurology* 2001; **57**: 920–4.

14 Reilmann R, Bohlen S, Klopstock T, *et al.* Grasping premanifest Huntington's disease – shaping new endpoints for new trials. *Mov Disord* 2010; **25**: 2858–62.

15 Tabrizi SJ, Scahill RI, Durr A, *et al.* Biological and clinical changes in premanifest and early stage Huntington's disease in the TRACK-HD study: the 12-month longitudinal analysis. *Lancet Neurol* 2011; **10**: 31–42.

16 Gountouna VE, Job DE, McIntosh AM, *et al.* Functional Magnetic Resonance Imaging (fMRI) reproducibility and variance components across visits and scanning sites with a finger tapping task. *Neuroimage* 2010; **49**: 552–60.

17 Gradin V, Gountouna VE, Waiter G, *et al.* Between- and within-scanner variability in the CaliBrain study n-back cognitive task. *Psychiatry Res* 2010; **184**: 86–95.

18 Yendiki A, Greve DN, Wallace S, *et al.* Multi-site characterization of an fMRI working memory paradigm: reliability of activation indices. *Neuroimage* 2010; **53**: 119–31.

19 Glover GH, Mueller BA, Turner JA, *et al.* Function biomedical informatics research network recommendations for prospective multicenter functional MRI studies. *J Magn Reson Imaging JMRI* 2012; **36**: 39–54.

20 Power JD, Barnes KA, Snyder AZ, Schlaggar BL, Petersen SE. Spurious but systematic correlations in functional connectivity MRI networks arise from subject motion. *NeuroImage* 2012; **59**: 2142–54.

21 Van Dijk KRA, Sabuncu MR, Buckner RL. The influence of head motion on intrinsic functional connectivity MRI. *NeuroImage* 2012; **59**: 431–8.

22 Power JD, Mitra A, Laumann TO, Snyder AZ, Schlaggar BL, Petersen SE. Methods to detect, characterize, and remove motion artifact in resting state fMRI. *NeuroImage* 2014; **84**: 320–41.

23 Saad ZS, Gotts SJ, Murphy K, *et al.* Trouble at Rest: How Correlation Patterns and Group Differences Become Distorted After Global Signal Regression. *Brain Connect* 2012; **2**: 25–32.

24 Fox MD, Zhang D, Snyder AZ, Raichle ME. The global signal and observed anticorrelated resting state brain networks. *J Neurophysiol* 2009; **101**: 3270–83.

25 Klöppel S, Draganski B, Siebner HR, Tabrizi SJ, Weiller C, Frackowiak RSJ. Functional Compensation of Motor Function in Pre-Symptomatic Huntington's Disease. *Brain* 2009; **132**: 1624–32.

26 Owen AM, McMillan KM, Laird AR, Bullmore E. N-back working memory paradigm: A meta-analysis of normative functional neuroimaging studies. *Hum Brain Mapp* 2005; **25**: 46–59.
